# Supplementary figures and images for: Social capital, social cohesion, and health of Syrian refugee working children living in informal tented settlements in Lebanon: A cross-sectional study
Source: PLoS Med. 2020 Sep 2;17(9):e1003283. doi: 10.1371/journal.pmed.1003283 (PMC7467280; doi:10.1371/journal.pmed.1003283)

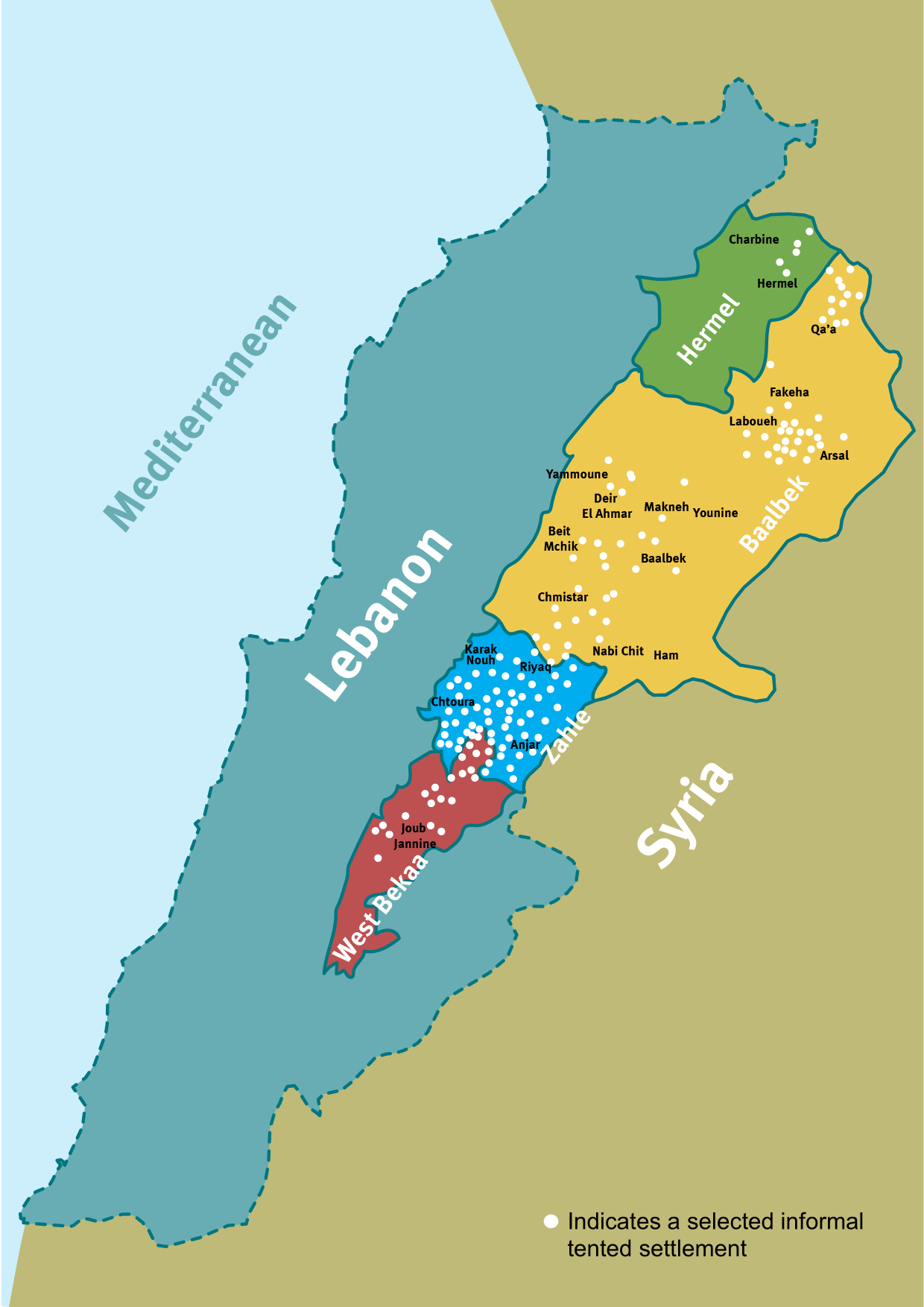

Supplement: S1 Fig — ITS, informal tented settlement (TIFF) [file pmed.1003283.s001.tiff]
