# Supplementary material for: Social capital, social cohesion, and health of Syrian refugee working children living in informal tented settlements in Lebanon: A cross-sectional study
Source: PLoS Med. 2020 Sep 2;17(9):e1003283. doi: 10.1371/journal.pmed.1003283 (PMC7467280; doi:10.1371/journal.pmed.1003283)
Supplement: S1 Text — (DOCX) [file pmed.1003283.s002.docx]

**S1 Text. Study Protocol**

**Survey on child labor in agriculture in the Beqaa valley of Lebanon: The case of Syrian refugees**

**Rima R. Habib and Collaborators**

**AUB/FHS research team**

[**rima.habib@aub.edu.lb**](mailto:rima.habib@aub.edu.lb)

**Study Protocol - January 2016**

**Introduction**

This study protocol offers a starting point for an assessment of child labor practices of displaced Syrians in agricultural settings in Lebanon. The outbreak of the Syrian war in 2011 has had many implications on Lebanon’s precarious infrastructures and labor economy (Gebara, 2015). Up to date, more than one million Syrians are believed to have sought refuge in Lebanon and are registered with UNHCR (UNHCR, 2015). Data and information on the Syrians in Lebanon may become less accurate with the recent decision of the Lebanese government to stop the registration of new refugees. Recent studies have highlighted the poor living conditions of displacement for Syrians with serious difficulties of access to housing, healthy neighborhoods, clean food and water, education, and job opportunities (Habib et al, 2016). While the majority of the displaced live in urban settings in Lebanon, there is a large number of Syrians—mostly new and former seasonal workers and their families—who continues to live in Informal Tented Settlements (ITS) that are widely spread across agricultural settings in Lebanon (Dhala,2014; Lebanon Support, 2016).

The tense political and economic climate in Lebanon have further affected the daily conditions of Syrian refugees and contributed to changes in the division of labor within the families of the displaced. Since the beginning of the Syrian refugees crisis, Lebanese lawmakers have banned Syrian adults (16 years and older) from the labor market with harsh punitive fines to employers and Syrian adults who break this law. Given the history of Syrian agricultural labor migration into Lebanon that preceded the crisis, such laws had drastic effects on the Lebanese agriculture landowners and the displaced Syrians. Specifically, many Syrian families and Lebanese employers have become dependent on Syrian children (who represent half of the four million of displaced Syrians across the region), especially in the agriculture sector. In Lebanon, Syrian children have increasingly entered the workforce to provide for their families and close the gap of shortage of agricultural labor force in the country.

Reports from Syria, Turkey, Jordan, and Lebanon indicate a trend of widespread child labor among the Syrian refugees (Demir et al, 2006; Hawamdeh and Spencer, 2002). In Syria, three-quarters of all school-aged children are part- or sole-breadwinners for their families compared to about 50% in Jordan. Similarly, nearly three-quarters of the Syrian children in Turkey are not enrolled in schools, which is suggestive of their involvement in the labor market (Human Rights Watch, 2015). Showing the intertwined relationship between agricultural labor and displacement in Lebanon, recent reports have highlighted the increasing numbers of Syrian children working in Lebanese agricultural settings in the North and the Beqaa Valley. A “*Situation analysis of youth in light of the Syrian crisis”* (UNICEF et al, 2014) showed that more than half of the young displaced Syrians are employed, with around 45 percent as daily and/or seasonal workers in the sectors that have traditionally used Syrian labor, such as agriculture. It has been suggested that children as young as six years old are working for 5-10 hour shifts and earning less than $1 per hour (Shaheen, 2015, 25 July). A recent study conducted by the UNICEF on child laborer practices in the Beqaa valley has highlighted a negative relationship between child labor and education. The study showed that nearly 36% of Syrian children working in agriculture were functionally illiterate—that is with inadequate reading and writing skills to manage daily living and employment tasks (Save the Children & UNICEF, 2015). Research and anecdotal evidence also suggest that many of these children are involved in hazardous and exploitative working conditions. The prevalence of child labor in agriculture has reportedly substantially increased (MOL and ILO, 2016) exposing children to worrisome occupational safety, health risks and social abuse. Child workers are regularly exposed to pesticides, toxic chemicals, heavy loads, and long-work-hours (Shaheen, 2015, 25 July).

This phenomenon requires urgent attention from national authorities and international agencies to curb child labor and consider intervention and coping strategies, including but not limited to reducing exposure of children to hazards at work, availing formal or informal education to all children, and facilitating income-generating activities for adults**.** Such measures nonetheless require a full understanding on the dynamics of child labor and its social, political, economic, and cultural contexts.

In light of the above, multiple UN agencies (ILO, UNICEF, FAO) and the Lebanese Ministry of Labor approached the Faculty of Health Sciences (FHS) at the American University of Beirut to conduct a study to better understand Syrian children’s engagement and practices in agricultural work and their working conditions and to recommend policies and action that can be effective in protecting children from worst forms of child labor and consider agricultural and other projects that lead to minimal school dropout.

**Objectives**

The objective of this study is to explore child labor conditions and practices in select agricultural settings in Beqaa, Lebanon and to determine the nature and dynamics of the precarious involvement of Syrian children in the Lebanese labor force. Building on an inter-disciplinary approach, the study aims to document the multifaceted consequences of child labor on health, wellbeing, and rights of the children further contributing to a better understanding and analysis of the Syrian refugee situation in Lebanon, and in particular child labor.

Specifically, the FHS research team seeks to answer the following questions:

1. What are the socio-economic and cultural contexts in which Syrian refugees children work?
2. What are the leading factors and consequences of child labor on the health and wellbeing of the children themselves, their households and communities?
3. How does schooling and education opportunities impact child labor? How has child labor affected schooling practices and education in the displacement settings?
4. How can the living and work environments be improved for the children and their families?

The FHS team will also produce a comprehensive report in English. Disseminating the findings to a wide range of stakeholders, translating the report into Arabic, and/or transforming some of the findings into guidelines, brochures, or other awareness material is not part of the current project.

**Data Collection Instruments and Methods**

The study employs a methodology to engage with the questions raised above. This includes: a desk review and document analysis and a quantitative survey developed for the assessment for child labor practices among the displaced children and their families. Ethical approval will be sought from the Institutional Review Board (IRB) at AUB. This will include developing, with care, consent and assent forms that will be required for the research engagement with a vulnerable population and to ensure ethical research conduct. The above-mentioned methodological approaches are described below.

1. **Desk Review and Documents Analysis**

The study team will carry out identification and analysis of available studies, statistics, and grey literature regarding child labor among Syrian refugees in Lebanon and the region. This includes social, economic, and health studies, surveys, news articles, and reports by international and local organizations. Statistical data will be collected from different sources with a particular focus on the agricultural sector. Data will also be gathered from national surveys in Lebanon that are particularly valuable as they contain information regarding work done by households and their various members. This activity will also include an examination of the history of legal frameworks that defined the work of Syrians in Lebanon, the legal frameworks relevant to child labor in Lebanon, and the enforcement of laws and rulings at the local and regional levels.

1. **Household Assessment**

A household survey with participating families in Syrian refugee communities located near agricultural areas will be conducted. The structured questionnaires will include a *household and parent* questionnaire and a *child* questionnaire. The household questionnaire will collect data on household demographics, education, migration history, employment history, social capital and family assets, availability of/access to/use of services, and social and family practices. The child questionnaire will collect data on conditions of life and work, specifically child workers demographics, work history and experience, education, health status and experiences, and perceived outlook on life.

**Data analysis**

The quantitative data from the survey will be entered into a descriptive analysis aimed at identifying trends in the population data. Findings will be categorized by age, gender, district, and other variables, when relevant.

**Timeline and deliverables**

The project will commence on January 15, 2016 and extend for 18 months. In the first four months, study tools and instruments will be developed, the sample of households to be visited will be identified, and AUB’s IRB clearance will be secured. In parallel, fieldworkers will be identified and trained in a classroom setting and in the field (pilot study). Data collection and entry will be followed by data analysis and reporting.

By the end of the study, AUB-FHS’ research team will submit a report on the main findings of the study with a focus on the conditions of life and work for Syrian working children, socioeconomic processes and environment within which children work, and will suggest recommendations and guidelines for intervention at the field, family, community, and policy levels. The FHS team is ready to give a public presentation on the main findings of the study to a wide audience invited by the UN agencies and MOL at the end of the project at a time convenient to all engaged parties.

**References**

Dhala, K. (2014). Forgotten camps: Syrian refugees in Lebanon’s Bekaa valley.

Amnesty International. Retrieved from <https://bit.ly/2O351Vr>

Demir, C. E., Demir, E., & Uygur, S. (2006). Work Academic Performance and School Attendance of Primary School Children in Turkey. Paper presented at the European Conference on Educational Research, Geneva

Gebara, K. (2015). The Syrian Crisis & its Implications on Lebanon. Social, Economic,

Political and Security Challenges an Potential Solutions [PowerPoint slides].

Retrieved from <https://bit.ly/2NjKC2f>

Hawamdeh, H., & Spencer, N. (2002). *Growth of working boys in Jordan: a crosssectional survey using non-working male siblings as comparisons.* Child: Care, Health and Development, 28(1), 47-49.

Habib, R. R., Mikati, D., Hojeij, S., El Asmar, K., Chaaya, M., & Zurayk, R. (2016). *Associations between poor living conditions and multi-morbidity among Syrian migrant agricultural workers in Lebanon.* The European Journal of Public Health, 26(6), 1039-1044. doi:10.1093/eurpub/ckw096

Human Rights Watch, 2015: <https://www.hrw.org/news/2015/11/08/turkey-400000-syrian-children-not-school>

Lebanon Support. (2016). Syrian Refugees’ Livelihoods. *The Impact of Progressively Constrained Legislations and Increased Informality on Syrians’ Daily Lives.* Retrieved from <https://bit.ly/2jOAkpo>

Ministry of Labor and ILO, *National Action Plan to Eliminate the Worst Forms of Child Labor in Lebanon by 2016*. Available from <http://www.ilo.org/beirut/publications/WCMS_229103/lang--en/index.htm>

Save the Children & UNICEF, 2015: https://gallery.mailchimp.com/5eb1a46c176e09c237df0913f/files/CHILD_LABOR.pdf

Shaheen, 2015: [http://www.theguardian.com/world/2015/jul/26/children-syria-beqaa-isis](http://www.theguardian.com/world/2015/jul/26/children-syria-bekaa-isis)

UNHCR, 2015 http://data.unhcr.org/syrianrefugees/country.php?id=122

UNICEF, UNFPA, UNESCO, UNHCR and SCI, *Situation analysis of youth in light of the Syrian crisis*, 2014. Available from <http://www.unfpa.org.lb/Publications.aspx>
