# Supplementary material for: Social capital, social cohesion, and health of Syrian refugee working children living in informal tented settlements in Lebanon: A cross-sectional study
Source: PLoS Med. 2020 Sep 2;17(9):e1003283. doi: 10.1371/journal.pmed.1003283 (PMC7467280; doi:10.1371/journal.pmed.1003283)
Supplement: S2 Text — (DOCX) [file pmed.1003283.s003.docx]

**S2 Text. Questions and Answer Choices**

| **Questions** | **Answer choices** |
| --- | --- |
| **Sociodemographic characteristics** | |
| What is your monthly salary? [Income/month] | Enter an integer |
| District name | 1. Baalbek  2. Hermel  3. West Bekaa  4. Zahle |
| Sex | 1. Male  2. Female |
| Household ID | Enter an integer |
| How old are you? [Age] | Enter an integer |
| Gender | 1. Male  2. Female |
| **Work Information** |  |
| In which field do you work? | Multiple options allowed |
| Agriculture | 0. No  1. Yes |
| Waste picking | 0. No  1. Yes |
| Construction | 0. No  1. Yes |
| Car wash | 0. No  1. Yes |
| Street services | 0. No  1. Yes |
| Factory employees | 0. No  1. Yes |
| Mechanics | 0. No  1. Yes |
| Other field of work | 0. No  1. Yes |
| How many hours/day do you work? | Enter an integer (hours/day)  98. No answer |
| **Health Status** |  |
| How do you compare your health to others of your age? | 1. Very Good  2. Good  3. Average  4. Poor  5. Very poor  98. No answer  99. I don’t know |
| Self-rated health | 0. Good (Very Good & Good)  1. Poor (Average, Poor & Very poor) |
| Do you suffer from any health problems? | 1. Yes  2. No  98. No answer  99. I don’t know |
| How do you identify yourself regarding smoking cigarettes? | 1. Cigarettes smoker  2. Tried it but never liked it  3. Used to smoke  4. Non cigarettes smoker  98. No answer |
| How do you identify yourself regarding smoking waterpipe? | 1. Waterpipe smoker  2. Tried it but never liked it  3. Used to smoke  4. Non waterpipe smoker  98. No answer |
| In the past 30 days in your leisure time (i.e. after school and during weekends), did you actively participate in sports or exercising? | 0. No  1. Yes |
| Risky health behaviors (smoking/ physical inactivity) | 0. No  1. Yes |
| **Feelings of loneliness** | |
| How often do you feel lonely and wish you had more friends? | 1. Often  2. Sometimes  3. Hardly ever  4. Never  98. |
| Feel lonely | 0. No  1.Yes |
| **Child optimism**  A child optimism index was developed using the following 10 items | |
| In uncertain times, I usually expect the best | 1. Agree  2. Disagree  3. Don’t know |
| It is easy for me to relax | 1. Agree  2. Disagree  3. Don’t know |
| If something can go wrong for me, it will happen | 1. Agree  2. Disagree  3. Don’t know |
| I'm always optimistic about my future | 1. Agree  2. Disagree  3. Don’t know |
| I enjoy my time with friends a lot | 1. Agree  2. Disagree  3. Don’t know |
| It's important for me to keep busy | 1. Agree  2. Disagree  3. Don’t know |
| I hardly ever expect things to go my way | 1. Agree  2. Disagree  3. Don’t know |
| I do not get upset too easily | 1. Agree  2. Disagree  3. Don’t know |
| I rarely expect good things to happen | 1. Agree  2. Disagree  3. Don’t know |
| Generally, I expect more good things to happen to me than bad things | 1. Agree  2. Disagree  3. Don’t know |
| Feel Optimistic | 1. Agree  0. Disagree |
| **Child satisfaction with life**  A child life satisfaction index was developed using the following 12 items:  How satisfied are you with | |
| the quality of your education at school? | 1. Very Satisfied  2. Satisfied  3. Not so satisfied  4. Not satisfied at all  5. Not applicable |
| your relationship with your teachers? | 1. Very Satisfied  2. Satisfied  3. Not so satisfied  4. Not satisfied at all  5. Not applicable |
| the financial situation of your family? | 1. Very Satisfied  2. Satisfied  3. Not so satisfied  4. Not satisfied at all  5. Not applicable |
| your health? | 1. Very Satisfied  2. Satisfied  3. Not so satisfied  4. Not satisfied at all  5. Not applicable |
| your weight? | 1. Very Satisfied  2. Satisfied  3. Not so satisfied  4. Not satisfied at all  5. Not applicable |
| your life? | 1. Very Satisfied  2. Satisfied  3. Not so satisfied  4. Not satisfied at all  5. Not applicable |
| your school? | 1. Very Satisfied  2. Satisfied  3. Not so satisfied  4. Not satisfied at all  5. Not applicable |
| future study opportunities? | 1. Very Satisfied  2. Satisfied  3. Not so satisfied  4. Not satisfied at all  5. Not applicable |
| future work opportunities? | 1. Very Satisfied  2. Satisfied  3. Not so satisfied  4. Not satisfied at all  5. Not applicable |
| leisure time opportunities? | 1. Very Satisfied  2. Satisfied  3. Not so satisfied  4. Not satisfied at all  5. Not applicable |
| opportunities to take part in social/ activities? | 1. Very Satisfied  2. Satisfied  3. Not so satisfied  4. Not satisfied at all  5. Not applicable |
| opportunities to voice your opinion in your school or community about issues that affect young people? | 1. Very Satisfied  2. Satisfied  3. Not so satisfied  4. Not satisfied at all  5. Not applicable |
| Satisfied with life | 1. Agree  0. Disagree |
| **Social cohesion** |  |
| *Connectedness* |  |
| In the past 30 days in your leisure time (i.e. after school and during weekends), did you spend time with friends? | 0. No  1. Yes |
| In the past 30 days in your leisure time (i.e. after school and during weekends), did you go around with friends just for fun (To places like streets, parks, etc...) | 0. No  1. Yes |
| Do you agree with this statement: “In general, one must be cautious when dealing with other people”? | 0. No  1. Yes |
| **Social capital** | |
| *Social support* | |
| If you had a personal problem, would you turn to someone for help? | 1. No  2. Yes |
| How is your relationship with your parents/siblings? | 1. Good  2. Poor |
| *Social leverage* | |
| Do you know of any nearby organization/association that provides services and help for refugees? | 1. Yes  2. No  98. No answer |
| Do you currently go to school in Lebanon? | 1. Yes  2. No |
| In which grade are you now? | Enter an integer |
| Do you take classes outside school? | 1. Yes  2. No |
| *Informal social control* | |
| Do you feel safe walking down your street after dark? | 0. No  1. Yes |
| *Neighbourhood organization participation* | |
| In the past 30 days in your leisure time (i.e. after school and during weekends), did you do volunteer work? | 0. No  1. Yes |
| *Family social capital* | |
| Do your parents (mom and/or dad) talk over important family decisions with you? | 0. No  1. Yes |
| Do your parents (mom and/or  dad) talk over your important  personal decisions with you? | 0. No  1. Yes |
| **Neighborhood attachment** | |
| Do you have close friend whom you can trust? | 0. No  1. Yes |
