# Supplementary material for: Social capital, social cohesion, and health of Syrian refugee working children living in informal tented settlements in Lebanon: A cross-sectional study
Source: PLoS Med. 2020 Sep 2;17(9):e1003283. doi: 10.1371/journal.pmed.1003283 (PMC7467280; doi:10.1371/journal.pmed.1003283)
Supplement: S1 Table — (DOCX) [file pmed.1003283.s005.docx]

**S1 Table. Unadjusted Odds Ratios corresponding to Table 2.**

**Unadjusted odds ratios for the associations between social cohesion, social capital and physical health for working children (8-18 years) in 1,902 Syrian refugee households living in informal tented settlements, Bekaa, Lebanon, 2017 (N=4,090)^a^.**

|  | Poor Self-rated health  (N=442, 10.81%) | Reported a health problem  (N=632, 15.46%) | Engaged in risky health behaviors (smoking/ physical inactivity)  (N=535, 13.08 %) |
| --- | --- | --- | --- |
|  | **Unadjusted OR^b^ (95% CI^c^)(p-value)** | **Unadjusted OR (95% CI)(p-value)** | **Unadjusted OR (95% CI)(p-value)** |
|  |  |  |  |
| Socioeconomic characteristics |  |  |  |
| Mean income (USD^d^) (log) | 0.86 (0.78- 0.94)(0.001) | 0.8 (0.76-0.89)(<0.001) | 1.15 (1.04- 1.26)(0.006) |
|  |  |  |  |
| Social cohesion |  |  |  |
| *Connectedness* |  |  |  |
| Spend time with friends |  |  |  |
| -Yes (ref) | 1 | 1 | 1 |
| -No | 2.6 (2.07-3.24)(<0.001) | 18 (1.46- 2.21)(<0.001) | 0.44 (0.32- 0.61)(<0.001) |
|  |  |  |  |
| Have fun with friends |  |  |  |
| -Yes (ref) | 1 | 1 | 1 |
| -No | 1.36 (0.83- 2.22)(0.22) | 1.1 (0.75- 1.63)(0.62) | 0.1 (0.10- 0.18)(<0.001) |
|  |  |  |  |
| Social capital |  |  |  |
| *Social support* |  |  |  |
| Have someone to consult with on personal problems |  |  |  |
| -Yes (ref) | 1 | 1 | 1 |
| -No | 1.2 (0.88- 1.60)(0.24) | 1.1 (0.85-1.43)(0.44) | 1.6 (1.26-2.08)(<0.001) |
|  |  |  |  |
| *Social leverage* |  |  |  |
| Know aid organizations |  |  |  |
| -Yes (ref) | 1 | 1 | 1 |
| -No | 0.6 (0.45-0.90)(0.01) | 0.79 (0.57- 1.09)(0.15) | 0.3 (0.24- 0.43)(<0.001) |
|  |  |  |  |
| *Informal social control* |  |  |  |
| Feel safe in street after dark |  |  |  |
| -Yes (ref) | 1 | 1 | 1 |
| -No | 1.9 (1.56-2.33)(<0.001) | 1.31 (1.11- 1.55)(0.002) | 0.9 (0.76- 1.10)(0.33) |
|  |  |  |  |
| *Neighbourhood organization participation* |  |  |  |
| Do volunteer work |  |  |  |
| -Yes (ref) | 1 | 1 | 1 |
| -No | 1.0 (0.74-1.36)(0.98) | 0.99 (0.76- 1.28)(0.94) | 0.3 (0.25- 0.38)(<0.001) |
|  |  |  |  |
| *Family social capital* |  |  |  |
| Discuss family issues with parents |  |  |  |
| -Good (ref) | 1 | 1 | 1 |
| -Poor | 1.9 (1.57-2.34)(<0.001) | 1.5 (1.23-1.73)(<0.001) | 1.8 (1.49- 2.15)(<0.001) |
|  |  |  |  |
| Neighborhood attachment |  |  |  |
| Have a close friend in the neighborhood |  |  |  |
| -Yes (ref) | 1 | 1 | 1 |
| -No | 1.4 (1.14- 1.78)(0.002) | 1.16 (0.95- 1.42)(0.14) | 0.6 (0.43- 0.71)(<0.001) |

^a^ Model clustered at household level with unadjusted odds ratios.

^b^ Unadjusted Odds Ratio.

^c^ Confidence Interval.

^d^ United States Dollar.
