# Supplementary material for: Social capital, social cohesion, and health of Syrian refugee working children living in informal tented settlements in Lebanon: A cross-sectional study
Source: PLoS Med. 2020 Sep 2;17(9):e1003283. doi: 10.1371/journal.pmed.1003283 (PMC7467280; doi:10.1371/journal.pmed.1003283)
Supplement: S2 Table — (DOCX) [file pmed.1003283.s006.docx]

**S2 Table. Unadjusted Odds Ratios corresponding to Table 3.**

**Unadjusted odds ratios for the associations between social cohesion, social capital and emotional wellbeing for working children (8-18 years) in 1,902 Syrian refugee households living in informal tented settlements, Bekaa, Lebanon, 2017 (N=4,090)^a^.**

|  | More lonely  (N=2761, 67.52%) | More optimistic (N=2154, 52.66%) | More satisfied with life (N=2396, 58.58%) |
| --- | --- | --- | --- |
|  | **Unadjusted OR^b^ (95% CI^c^)(p-value)** | **Unadjusted OR (95% CI)(p-value)** | **Unadjusted OR (95% CI)(p-value)** |
|  |  |  |  |
| Socioeconomic characteristics |  |  |  |
| Mean income (USD^c^) (log) | 1.2 (1.10- 1.25)(<0.001) | 1.02 (0.97-1.09)(0.41) | 0.73 (0.68- 0.78)(<0.001) |
|  |  |  |  |
| Social cohesion |  |  |  |
| *Connectedness* |  |  |  |
| Spend time with friends |  |  |  |
| -No (ref) | 1 | 1 | 1 |
| -Yes | 0.4 (0.33-0.56)(<0.001) | 1.01 (0.77-1.33)(0.92) | 1.24 (0.94- 1.66)(0.12) |
|  |  |  |  |
| Cautious when dealing with other people |  |  |  |
| -No (ref) | 1 | 1 | 1 |
| -Yes | 2.68 (1.70- 4.23)(<0.001) | 1.2 (0.77- 1.89)(0.42) | 0.9 (0.60- 1.51)(0.83) |
|  |  |  |  |
| Social capital |  |  |  |
| *Social support* |  |  |  |
| Have someone to consult with on personal problems |  |  |  |
| -No (ref) | 1 | 1 | 1 |
| -Yes | 0.9 (0.77- 1.16)(0.6) | 2.1 (1.69-2.51)(<0.001) | 1.9 (1.60-2.36)(<0.001) |
| Quality of social relations |  |  |  |
| -Poor (ref) | 1 | 1 | 1 |
| -Good | 0.8 (0.66-0.89)(<0.001) | 1.44 (1.24-1.66)(<0.001) | 1.41 (1.22-1.64)(<0.001) |
|  |  |  |  |
| *Social leverage* |  |  |  |
| Going to school |  |  |  |
| -No (ref) | 1 | 1 | 1 |
| -Yes | 0.5 (0.42-0.58)(<0.001) | 1.0 (0.0.86- 1.19)(0.92) | 6.9 (5.47- 8.87)(<0.001) |
|  |  |  |  |
| Taking classes outside school |  |  |  |
| -No (ref) | 1 | 1 | 1 |
| -Yes | 0.3 (0.22-0.53)(<0.001) | 1.3 (0.84-2.06)(0.23) | 2.7 (1.58-4.64)(<0.001) |
|  |  |  |  |
| Know of aid organizations |  |  |  |
| -No (ref) | 1 | 1 | 1 |
| -Yes | 0.4 (0.34-0.57)(<0.001) | 0.97 (0.75-1.24)(0.79) | 1.5 (1.17-1.99)(0.002) |
|  |  |  |  |
| *Informal social control* |  |  |  |
| Feel safe in street after dark |  |  |  |
| -No (ref) | 1 | 1 | 1 |
| -Yes | 1.2 (1.08-1.40)(0.002) | 0.9 (0.87- 1.11)(0.80) | 0.83 (0.73- 0.94)(0.003) |
|  |  |  |  |
| *Neighbourhood organization participation* |  |  |  |
| Do volunteer work |  |  |  |
| -No (ref) | 1 | 1 | 1 |
| -Yes | 0.7 (0.57-0.84)(<0.001) | 1.78 (1.46- 2.16)(<0.001) | 0.9 (0.76- 1.11)(0.36) |
|  |  |  |  |
| *Family social capital* |  |  |  |
| Discuss personal issues with parents |  |  |  |
| - Poor (ref) | 1 | 1 | 1 |
| -Good | 0.9 (0.74-0.97)(0.017) | 1.2 (1.06-1.36)(0.003) | 1.3 (1.13-1.45)(<0.001) |
|  |  |  |  |
| Neighborhood attachment |  |  |  |
| Have a close friend in the neighborhood |  |  |  |
| -No (ref) | 1 | 1 | 1 |
| -Yes | 0.5 (0.41-0.58)(<0.001) | 2.1 (1.80- 2.44)(<0.001) | 1.5 (1.26- 1.70)(<0.001) |

^a^ Model clustered at household level with unadjusted odds ratios.

^b^ Unadjusted Odds Ratio.

^c^ Confidence Interval.

^d^ United States Dollar.
