# Supplementary material for: Social capital, social cohesion, and health of Syrian refugee working children living in informal tented settlements in Lebanon: A cross-sectional study
Source: PLoS Med. 2020 Sep 2;17(9):e1003283. doi: 10.1371/journal.pmed.1003283 (PMC7467280; doi:10.1371/journal.pmed.1003283)
Supplement: Attachment — Submitted filename: Draft10-Response to Reviewers-SocialCapital-7May20.docx [file pmed.1003283.s007.docx]

**REQUESTS FROM THE EDITORS**

**Comment 1:** Data Availability Statement: The Data Availability Statement (DAS) requires revision. Thanks for noting that the data are contained within the manuscript. PLOS Medicine requires that the de-identified data underlying the specific results in a published article be made available, without restrictions on access, in a public repository or as Supporting Information at the time of article publication, provided it is legal and ethical to do so. Please see the policy at

<https://eur03.safelinks.protection.outlook.com/?url=http%3A%2F%2Fjournals.plos.org%2Fplosmedicine%2Fs%2Fdata-availability&amp;data=02%7C01%7Crima.habib%40aub.edu.lb%7C492ad8a2fc5c446fe18308d7dfd2d21e%7Cc7ba5b1a41b643e9a1206ff654ada137%7C1%7C0%7C637223968875364214&amp;sdata=BijTMKH%2BqrTDw%2FkALqLC%2Fii8y7nQ6cNQtA6Hv3s%2BUL8%3D&amp;reserved=0>

and FAQs at

<https://eur03.safelinks.protection.outlook.com/?url=http%3A%2F%2Fjournals.plos.org%2Fplosmedicine%2Fs%2Fdata-availability%23loc-faqs-for-data-policy&amp;data=02%7C01%7Crima.habib%40aub.edu.lb%7C492ad8a2fc5c446fe18308d7dfd2d21e%7Cc7ba5b1a41b643e9a1206ff654ada137%7C1%7C0%7C637223968875364214&amp;sdata=20diuNUsg1vTjuNXcfJ0SoR7E8k00l0xs40f24wHq20%3D&amp;reserved=0>

Specifically, for each data source used in your study:

a) If the data are freely or publicly available, note this and state the location of the data: within the paper, in Supporting Information files, or in a public repository (include the DOI or accession number).

b) If the data are owned by a third party but freely available upon request, please note this and state the owner of the data set and contact information for data requests (web or email address). Note that a study author cannot be the contact person for the data.

c) If the data are not freely available, please describe briefly the ethical, legal, or contractual restriction that prevents you from sharing it. Please also include an appropriate contact (web or email address) for inquiries (again, this cannot be a study author).

**Author’s response**: For enquiries about the project and data, please contact Ms. H. Mansour at the Faculty of Health Sciences, American University of Beirut. Email: hm102@aub.edu.lb.

**Comment 2:** Title: Please revise your title according to PLOS Medicine's style. Please place the study design ("A randomized controlled trial," "A retrospective study," "A modelling study," etc.) in the subtitle (ie, after a colon).

**Author’s response**: We thank the editors for this comment. We have edited the study title as follows

Social Capital, Social Cohesion and Health of Syrian Refugee Working Children Living in Informal Tented Settlements in Lebanon: A Cross-Sectional Study

**Comment 3:** Abstract: Methods and Findings: Please clearly state the main outcome measures of interest in the study.

**Author’s response**: As per the editors’ request, we have added the main outcome measures of interest in the Methods and Findings section of the Abstract as follows:

Logistic regressions assessed the association of social capital and social cohesion with the health and emotional wellbeing of Syrian refugee working children; specifically, poor self-rated health, reporting a health problem, engaging in risky health behavior, feeling lonely, feeling optimistic, and being satisfied with life.

**Comment 4:** Abstract: Methods and Findings: Please quantify the main results for the reported associations with both 95% CIs and p values. Please define the abbreviation AOR at first use.

**Author’s response**: We have added the p values and defined the abbreviation AOR in the Methods and Findings section of the Abstract as follows:

Lower levels of social cohesion (e.g., not spending time with friends) were significantly associated with poor self-rated health, reporting a physical health problem and feeling more lonely [Adjusted Odds Ratio (AOR): 2.4, CI: 1.76-3.36, p<0.001), (AOR: 1.9, CI: 1.44-2.55, p<0.001) and (AOR: 0.5, CI: 0.38-0.76, p<0.001), respectively]. Higher levels of social support (e.g. having good social relations), family social capital (e.g. discussing personal issues with parents), and neighborhood attachment (e.g. having a close friend) were all significantly associated with being more optimistic [(AOR: 1.5, CI: 1.2-1.75, p<0.001), (AOR: 1.3, CI: 1.11-1.52, p<0.001) and (AOR: 1.9, CI: 1.58-2.29, p<0.001), respectively] and more satisfied with life [(AOR: 1.3, CI: 1.01-1.54, p<0.04), (AOR: 1.2, CI: 1.01-1.4, p<0.04) and (AOR: 1.3, CI: 1.08-1.6, p=0.006), respectively].

**Comment 5:** Abstract: Methods and Findings: Please include the important variables that are adjusted for in the analyses.

**Author’s response**: Thank you for this comment, we have added in the Methods of the Abstract the variables that we adjusted for in the analyses and corrected the inadvertent mistake in the footnotes of the tables.

**Comment 6:** Abstract: Methods and Findings: In the last sentence of the Abstract Methods and Findings section, please describe the main limitation(s) of the study's methodology.

**Author’s response**: We have added the following main study limitations to the Methods and Findings section of the Abstract:

The main limitations of this study were its cross-sectional design, using self-reported health measures, and giving equal weighting to all the components of the health and emotional wellbeing indicators.

**Comment 7:** Abstract: Conclusions: Please address the study implications without overreaching what can be concluded from the data; please revise the sentence "Multidimensional interventions that consider social capital dimensions are needed to improve the health of Syrian refugee children in informal tented settlements.” as it suggests a causal relationship between social capital and health, and your study cannot speak to a causal relationship.

**Author’s response**: We have revised the last sentence in the Conclusions section of the Abstract to remove any suggestions of a causal relationship between social capital and health. The sentence now reads as follows:

Interventions that consider social capital dimensions might contribute to improving the health of Syrian refugee children in informal tented settlements.

**Comment 8:** Author Summary: At this stage, we ask that you include a short, non-technical Author Summary of your research to make findings accessible to a wide audience that includes both scientists and non-scientists. The Author Summary should immediately follow the Abstract in your revised manuscript. This text is subject to editorial change and should be distinct from the scientific abstract. Please see our author guidelines for more information: <https://eur03.safelinks.protection.outlook.com/?url=https%3A%2F%2Fjournals.plos.org%2Fplosmedicine%2Fs%2Frevising-your-manuscript%23loc-author-summary&amp;data=02%7C01%7Crima.habib%40aub.edu.lb%7C492ad8a2fc5c446fe18308d7dfd2d21e%7Cc7ba5b1a41b643e9a1206ff654ada137%7C1%7C0%7C637223968875364214&amp;sdata=jKUkBFT3mGAUBsq2IKbWLH3FPIaO%2FH6%2BDUOz1I%2FRhA0%3D&amp;reserved=0>

**Author’s response**: We have added the following Author Summary after the Abstract:

**AUTHOR SUMMARY**

Why was this study done?

- The war in Syria has resulted in a large displacement of population to neighboring Lebanon.
- Many vulnerable displaced families are living in informal tented settlements and have no access to means of livelihood and resources.
- Child labor among these displaced families is high.
- This study aimed to investigate the associations between social capital and cohesion and the health and emotional wellbeing of Syrian refugee children.
- Results from this study help inform policies and strategies that improve the wellbeing of these child refugees.

What did the researchers do and find?

- We conducted a cross-sectional study of 1,902 households of Syrian displaced families living in informal tented settlements in a rural area in Lebanon.
- We interviewed 4,090 working Syrian refugee children aged between 8 and 18 years.
- Around 11% of the working children rated their health as poor, 16% reported having a physical health problem, and 13% were engaged in risky health behaviors.
- Lower social cohesion was significantly associated with reporting poor health.
- A lower level of social support was significantly associated with engaging in risky health behaviors.
- Higher levels of neighborhood attachment, family social capital, and social support were significantly associated with greater optimism and life satisfaction.
- Higher levels of neighborhood attachment and social cohesion were significantly associated with feeling less lonely.

What do these findings mean?

- Social capital and social cohesion are associated with reporting better health and emotional wellbeing among Syrian refugee children working and living in Lebanon.
- Policies aimed at promoting the physical and emotional wellbeing of Syrian refugee children should consider interventions that aim to maintain social capital among these populations.

**Comment 9:** Introduction, paragraph 3 (and throughout): Your study is observational and therefore causality cannot be inferred. Please remove language that implies causality, such as “The current study addresses this gap in the literature by exploring social capital and social cohesion and their effects on the health of Syrian refugee children living and working in Lebanon.”

We suggest: “The current study addresses this gap in the literature by exploring the relationships between social capital and cohesion and the health of Syrian refugee children living and working in Lebanon.” or similar.

**Author’s response**: We have replaced the sentence in paragraph 3 of the Introduction with the sentence suggested by the editors. Also, we have revised the paper and edited any language that implies causality. The following sentences were edited:

In Abstract

In spite of the poor living and working conditions that Syrian refugee children experience, ~~those who enjoy~~ having a closely-knit network of family and friends ~~have experienced~~ was associated with better health ~~than those who did not~~. ~~Multidimensional~~ Interventions that consider social capital dimensions might contribute to ~~are needed to~~ improving the health of Syrian refugee children in informal tented settlements.

In Introduction

The current study addresses this gap in the literature by exploring the relationships between social capital and cohesion and ~~their effects on~~ the health of Syrian refugee children living and working in Lebanon.

In Discussion

Although higher income may translate to greater access to health-supporting resources and improving health ~~outcomes~~, it also implies that children are spending more time at work and hence less time with friends, which can lead to greater loneliness and less life satisfaction [56].

This is complemented by the finding that being cautious and consequently less socially connected is associated with an increase in the children’s loneliness and social isolation.

Poor relationships with the parents were associated with poorer health and wellbeing of the refugee children and ~~reduced their~~ lower optimism and satisfaction with life.

Neighborhood attachment, demonstrated through friendship, was an important factor ~~that affects~~ associated with health.

In Implications

In this study, we observed interconnections between social capital, social cohesion, and health. The social capital elements in this study ~~represented important factors in predicting~~ were associated with refugee ~~working~~ children’s health. We also highlight the critical role of social cohesion in establishing and maintaining social connectedness that is ~~reflected~~ related to ~~positive~~ improved health ~~outcomes~~.

**Comment 10:** Methods: Questionnaire: Please include the questionnaire and all the questions relating to outcome measures (e.g. social capital, social cohesion, neighborhood attachment, health behaviors, and health status), as well as a description of how answers were quantified, as a supporting information file, and refer to it in the text where the questionnaire is described.

**Author’s response**: The questionnaire was administered in colloquial Arabic. We translated the text in the questionnaires from colloquial Arabic to English and we provided the options that the respondents could choose from to provide a full description of the variables and data used in the analysis. The questionnaire is provided in supplementary file 3.

**Comment 11:** Results: Socioeconomic characteristics paragraph: Please provide the p values in addition to the 95% CIs associated with these analyses.

**Author’s response**: We have added the p values in the Socioeconomic characteristics paragraph of the Results:

The results showed that for every 10 USD increase in the mean income, children were at lower odds of reporting poor self-rated health (AOR: 0.81, CI: 0.73- 0.90, p<0.001) and reporting a health problem (AOR: 0.7, CI: 0.68-0.81, p<0.001). In contrast, for every 10 USD increase in the mean income, children were at higher odds of feeling lonely (AOR: 1.11, CI: 1.02-1.22, p=0.01) and at lower odds of life satisfaction (AOR: 0.88, CI: 0.80-0.96, p=0.005).

**Comment 12:** Results: Self-rated health and physical health problems paragraph: Please provide the p values in addition to the 95% CIs associated with these analyses.

**Author’s response**: We have added the p values in the Self-rated health and physical health problems paragraph of the Results:

Children who did not spend free time with their friends had higher odds of reporting poor self-rated health (AOR: 2.4, CI: 1.76-3.36, p<0.001) and a physical health problem (AOR: 1.9, CI: 1.44-2.55, p<0.001). Similarly, a lower level of informal social control, captured by not feeling safe in the street after dark, was significantly associated with poor perceived health. Children who reported feeling unsafe walking after dark were at higher odds of reporting poor self-rated health (AOR: 1.8, CI: 1.39-2.30, p<0.001). In addition, a lower level of family social capital (having poor relationships with the parents), was significantly associated with reporting poor health. Those who had poor relationships with their parents were at higher odds of reporting poor self-rated health (AOR: 1.6, CI: 1.22-2.00, p<0.001) and a physical health problem (AOR: 1.5, CI: 1.17-1.81, p<0.001), and were more likely to engage in risky behaviors (AOR: 1.6, CI: 1.29-2.09, p<0.001).

**Comment 13:** Results: Engagement in risky behaviors (smoking and physical inactivity): Please provide the p values in addition to the 95% CIs associated with these analyses.

**Author’s response**: We have added the p values in the Engagement in risky behaviors (smoking and physical inactivity) paragraph in the Results:

Children who did not have someone to consult with were more likely to engage in risky behaviors (AOR: 1.8, CI: 1.29-2.58, p<0.001).

Children who did not have a close friend in the neighborhood (neighborhood attachment) or did not engage in fun activities with friends in their free time (social cohesion) were less likely to engage in risky behaviors [(AOR: 0.7, CI: 0.49- 0.99, p=0.05) and (AOR: 0.3, CI: 0.19-0.39, p<0.001), respectively] (Table 2). In addition, children who were not familiar with aid organization (social leverage) or did not engage in voluntary work (neighborhood organization participation) were less likely to engage in risky behaviors [(AOR: 0.3, CI: 0.18-0.38, p<0.001) and (AOR: 0.5, CI: 0.37- 0.66, p<0.001), respectively].

**Comment 14:** Results: Feelings of loneliness paragraph: Please provide the p values in addition to the 95% CIs associated with these analyses.

**Author’s response**: We have added the p values in the Feelings of loneliness paragraph of the Results:

Children who had close friends in the neighborhood or who spent time with friends were at lower odds of feeling lonelier [(AOR: 0.5, CI: 0.39-0.62, p<0.001) and (AOR: 0.5, CI: 0.38-0.76, p<0.001), respectively].

Children who described the quality of their relations with others in their neighborhood as good or were aware of the presence of aid organization were at lower odds of feeling lonelier [(AOR: 0.8, CI: 0.63-0.96, p=0.016) and (AOR: 0.6, CI: 0.39-0.81, p=0.002), respectively]. Similarly, children who went to school or took classes outside school were at lower odds of feeling lonelier [(AOR: 0.6, CI: 0.48- 0.79, p<0.001) and (AOR: 0.4, CI: 0.22-0.76, p=0.005), respectively]. On the other hand, lower levels of social cohesions (being cautious when dealing with others) was significantly associated with feeling lonelier. Those who reported being cautious had higher odds of feeling lonelier (AOR: 3.4, CI: 1.95-5.84, p<0.001).

**Comment 15:** Results: Optimism and life satisfaction paragraph: Please provide the p values in addition to the 95% CIs associated with these analyses.

**Author’s response**: We have added the p values to the Optimism and life satisfaction paragraph in the Results:

Children who had good social relations or had a close friend in the neighborhood were at higher odds of reporting greater optimism [(AOR: 1.5, CI: 1.2-1.75, p<0.001) and (AOR: 1.9, CI: 1.58-2.29, p<0.001), respectively], and of being more satisfied with life [(AOR: 1.3, CI: 1.01-1.54, p<0.04) and (AOR: 1.3, CI: 1.08-1.6, p=0.006), respectively]. In addition, those who had someone to consult with on their personal problems or discussed their personal issues with their parents were at higher odds of reporting greater optimism [(AOR: 2, CI: 1.5-2.54, p<0.001) and (AOR: 1.3, CI: 1.11-1.52, p<0.001), respectively] and of being more satisfied with life [(AOR: 1.9, CI: 1.48-2.44, p<0.001) and (AOR: 1.2, CI: 1.01-1.4, p=0.04), respectively]. Similarly, higher levels of social leverage, measured by going to school, showed a significant association with being satisfied with life. Children who went to school were at higher odds reporting greater life satisfaction (AOR: 6.3, CI: 4.66-8.57, p<0.001).

**Comment 16:** Discussion, paragraph 6: Please revise the sentence as it implies causality: “Poor relationships with the parents affected the health and wellbeing of the refugee children and reduced their optimism and satisfaction with life.” We suggest: “Poor relationships with the parents were associated with poorer health and wellbeing of the refugee children and lower optimism and satisfaction with life.” or similar.

**Author’s response**: We have replaced the sentence in paragraph 6 of the Discussion section with the one suggested by the editors:

Poor relationships with the parents were associated with poorer health and emotional wellbeing of the refugee children and lower optimism and satisfaction with life.

**Comment 17:** Discussion, Implications paragraph: Throughout this paragraph, please address the study implications without overreaching what can be concluded from the data; the phrase "In this study, we observed ..." may be useful. Please revise or remove the sentence “The studied social capital elements represented important factors in predicting a refugee working child’s health.” as your results cannot speak to the predictive relationship between these elements, only that they were associated. Please similarly revise the rest of the paragraph to reduce causal implications of your study.

**Author’s response**: we have edited the Implications section of the Discussion in accordance with editors comments as follows

In this study, we observed interconnections between social capital, social cohesion, and health. The social capital elements in this study ~~represented important factors in predicting~~ were associated with refugee working children’s health. We also highlight the critical role of social cohesion in establishing and maintaining social connectedness that is ~~reflected~~ related to ~~positive~~ improved health ~~outcomes~~. It also highlights the importance of social capital in creating a ripple effect of promoting belonging and social inclusion. Considerations of social capital as a public policy tool to achieve social cohesion need to take into account the alternative conceptions of social capital rooted in an understanding of the refugees’ contexts, displacement and social interactions.

**Comment 18:** Table 1: It is confusing that standard deviation is presented in the percent column for some variables. In the legend, please define abbreviations for SD and USD.

**Author’s response**: We have edited Table 1 and separated the mean and standard deviation from the N and %. We also defined the abbreviations for SD and USD in the legend. Table 1 has been edited as follows:

**Table 1. Sociodemographic characteristics of the working children (>8-≤18 years) in 1,902 Syrian refugee households living in informal tented settlements, Bekaa, Lebanon, 2017 (N=4,090).**

| Characteristics | N | % |
| --- | --- | --- |
|  |  |  |
| ~~Age, years (Mean, SD)~~ | ~~13.2~~ | ~~2.7~~ |
|  |  |  |
| Age, years |  |  |
| 8-10 | 663 | 16.2 |
| 11-12 | 819 | 20 |
| 13-16 | 1860 | 45.5 |
| 17-18 | 748 | 18.3 |
|  |  |  |
| Gender |  |  |
| Male | 2107 | 51.5 |
| Female | 1983 | 48.5 |
|  |  |  |
| Field of work^a^ |  |  |
| Agriculture | 3098 | 75.8 |
| Waste picking | 173 | 4.2 |
| Construction | 147 | 3.6 |
| Car wash | 100 | 2.4 |
| Street services | 98 | 2.4 |
| Factory employees | 87 | 2.1 |
| Mechanics | 78 | 1.9 |
| Other^b^ | 382 | 9.3 |
|  |  |  |
| Going to school |  |  |
| Yes | 702 | 17.2 |
| No | 3388 | 82.8 |
|  |  |  |
| Child over age for grade^c,d^ |  |  |
| Yes | 589 | 83.9 |
| No | 113 | 16.1 |
|  |  |  |
|  | **Mean** | **SD^e^** |
|  |  |  |
| Number of children/household | 2.8 | 1.5 |
|  |  |  |
| Hours worked daily ~~(Mean, SD)~~ | 6.7 | 3 |
|  |  |  |
| Income/month, USD^f^ ~~(Mean, SD)~~ | 72.04 | 59.97 |

^a^ Total greater than 100% as more than one option is possible.

^b^ Includes handcrafts, packaging at markets, sewing, housekeeping, porting and other occupations.

^c^ Out of 702 children who go to school.

^d^ Based on age/school grade distribution in Lebanon: UNHCR & GoL. (2019). Education Sector. In: Lebanon Crisis Response Plan 2017-2020. Retrieved from:  <https://www.un.org.lb/library/assets/Education-015415.pdf>

^e^ Standard Deviation.

^f^ United States Dollar.

**Comment 19:** Table 2 and Table 3: Please provide the p values for these comparisons. Please provide the results from unadjusted analyses as well as the adjusted comparisons, and in the Table legend please define the abbreviations for AOR, CI and USD.

**Author’s response**: We have added the p values for the comparisons in Table 2 and Table 3. We have also defined the abbreviations for AOR, CI and USD in the legend for Table 2 and Table 3. We created separate tables for the unadjusted analyses which are included in the supplementary file (S4 Unadjusted Odds Ratios).

**Comment 20:** Checklist: Please ensure that the study is reported according to the STROBE guideline, and include the completed STROBE checklist as Supporting Information. When completing the checklist, please use section and paragraph numbers, rather than page numbers.

Please add the following statement, or similar, to the Methods: "This study is reported as per the Strengthening the Reporting of Observational Studies in Epidemiology (STROBE) guideline (S2 Checklist)."

**Author’s response**: We have completed a STROBE checklist and included it as Supporting Information. We have also added the proposed sentence to the Methods section as follows:

The study was approved by the Institutional Review Board at the American University of Beirut (IRB-RH1.08). This study is reported as per the Strengthening the Reporting of Observational Studies in Epidemiology (STROBE) guideline (S1 STROBE Checklist).

**Comment 21:** References: RE reference 51 listed as under review, papers cannot be listed in the reference list until they have been accepted for publication or are otherwise publicly accessible (for example, in a preprint archive). Please update reference 51. Please also in your response, note the advance that the current study provides over the study published in ref 51.

**Author’s response**: We have updated reference 51 as it is published now:

Habib RR, Ziadee M, Abi Younes E, El Asmar K, Jawad M. The association between living conditions and health among Syrian refugee children in informal tented settlements in Lebanon. Journal of Public Health. 2019. doi: <https://doi.org/10.1093/pubmed/fdz108>.

The above-mentioned paper focuses on the association between physical housing conditions and housing problems (such as, humidity, leakage, pest infestation, improper ventilation, inadequate heating and electricity supply) and the health of Syrian refugees. This study highlights the association between social capital dimensions (such as, quality of social relations, connectedness with friends and parents) and the health of Syrian refugee working children.

**REVIEWER 1 COMMENTS**

**Comment 1**: Although based on the conceptual model of social capital for health by Carpiano (2007), the survey questionnaire on social capital and cohesion has not been validated by any means therefore subject to scrutiny, which led to validity and reliability issues for the study.

**Author’s response**: We thank the reviewer for this comment. The survey questionnaire relating to social capital and cohesion was previously used by a research team (including the authors) in poor and marginalized communities living on the outskirts of Beirut, Lebanon [1]. The survey questionnaire has not been validated; however the questionnaire items are commonly used in the social capital and health literature. Also, during the pilot study phase, the investigators were reassured that the questions used measured the intended concepts.

1. Hajj TE, Afifi RA, Khawaja M, Harpham T. Violence and social capital among young men in Beirut. Injury Prevention. 2011;17(6):401-6.

**Comment 2**: The cross-section design of the study cannot offer any evidence on the causal relationships as hinted by authors in the abstract "those who enjoy a closely-knit network of family and friends have experienced better health than those who did not". It could be either way, low social networks led to poor health or poor health led to low social networks. Authors need to be very clear and strict on this.

**Author’s response**: We thank the reviewer for this comment. We have revised the paper and edited language that suggests a causal relationship between social capital, social cohesion and health. The following sentences were edited:

In Abstract

In spite of the poor living and working conditions that Syrian refugee children experience, ~~those who enjoy~~ having a closely-knit network of family and friends ~~have experienced~~ was associated with better health ~~than those who did not~~. ~~Multidimensional~~ Interventions that consider social capital dimensions might contribute to ~~are needed to~~ improving the health of Syrian refugee children in informal tented settlements.

In Introduction

The current study addresses this gap in the literature by exploring the relationships between social capital and cohesion and ~~their effects on~~ the health of Syrian refugee children living and working in Lebanon.

In Discussion

Although higher income may translate to greater access to health-supporting resources and improving health ~~outcomes~~, it also implies that children are spending more time at work and hence less time with friends, which can lead to greater loneliness and less life satisfaction [56].

This is complemented by the finding that being cautious and consequently less socially connected is associated with an increase in the children’s loneliness and social isolation.

Poor relationships with the parents were associated with poorer health and wellbeing of the refugee children and ~~reduced their~~ lower optimism and satisfaction with life.

Neighborhood attachment, demonstrated through friendship, was an important factor ~~that affects~~ associated with health.

In Implications

In this study, we observed interconnections between social capital, social cohesion, and health. The social capital elements in this study ~~represented important factors in predicting~~ were associated with refugee ~~working~~ children’s health. We also highlight the critical role of social cohesion in establishing and maintaining social connectedness that is ~~reflected~~ related to ~~positive~~ improved health ~~outcomes~~.

**Comment 3**: All the associations discovered in the study seem well-known already. Not sure what is the novelty of the study.

**Author’s response**: Although the associations between social capital and health have been well discussed in the literature, there is a dearth of research on the interplay between these factors among Syrian refugees in humanitarian settings. This paper focused on the interconnectedness between social capital dimensions and the health of Syrian refugee children. These relationships have not been well addressed among Syrian refugee children facing precarious living and working conditions, which is a main contribution of this study.

**REVIEWER 2 COMMENTS**

**Comment 1**: p. 3:  Many potential readers may not be familiar with the geographic setting. Authors may consider using a map (that in relation to sampling using IAMP might be even better).
Syrian refugees' livelihood might need more explanation as most refugees are not allowed to work in other contexts and (Syrian) refugees in Lebanon are in unique situations (e.g. not being called a "refugee" but "migrant"), which should be discussed for better contextual information.

**Author’s response**: We thank the reviewer for these comments. As per the reviewer’s request we have added a map as supplementary file 1 that we have produced in this project in relation to the sampling using IAMP. The map shows the spread of the selected informal tented settlements.

**Comment 2**: p.4: The way authors introduced social capital and social cohesion is quite abrupt. It would be nice to put more background and justification for introducing these concepts.

**Author’s response**: We thank the reviewer for this comment. We have edited this section in order to add more background and justification for introducing social capital and cohesion:

However, social capital and its relation to the health and wellbeing of refugees has not been thoroughly explored thus far. Given that Syrian refugees have been living in informal tented settlements in Lebanon for almost 9 years, their needs extend beyond daily necessities like food and shelter. Researchers need to give equal attention to the everyday relations that foster resilience and contribute to wellbeing within this refugee community. The current study addresses this gap in the literature by exploring the relationships between social capital and cohesion and ~~their effects on~~ the health of Syrian refugee children living and working in Lebanon.

**Comment 3**: P.5: The authors' own definition of social capital (based on the adopted conceptual model) should be demonstrated around here. The first paragraph of p.5 describes social cohesion as part of social capital and yet the figure separates them as distinct concepts, which is confusing.

**Author’s response**: We thank the reviewer for these remarks. We have added the definition of social capital adopted in this paper based on the conceptual model adopted:

This paper adopts Carpiano’s [49] conceptualization of social capital as the interaction between a group’s resources and the connections that individuals have to this group, which allow them to benefit from these resources.

Regarding social cohesion which is described as an aspect of social capital in the first paragraph of page 5, we would like to clarify that this is derived from the work of Araya et al. (2006). Carpiano, however, makes a distinction between social capital and social cohesion, hence the separate representation in the model (Figure 1). To avoid any possible confusion, we have omitted the terms “social cohesion” from the sentence as follows:

Previous studies showed a positive association between social capital and health [30-32]. Aspects of social capital that have been linked to better health include: trust (feeling safe in one’s neighborhood), ~~social cohesion (~~feelings of belonging and enjoying living in one’s neighborhood~~)~~ [33], informal social control [34], and community participation and neighborhood connections [31].

**Comment 4**: pp.6-9: How did the study deal with social desirability issues? The health measures include sensitive questions and the fieldworkers might happened to be acquaintances to those participants. Also, the age range is quite wide for some of these questions in avoiding measurement errors (e.g. significant gaps in capability and relevancy between nine and eighteen-year-old children in reporting loneliness and/or risky health behavior). More justification is necessary for adopting these questions.

Throughout the findings, authors should clearly mention any controlling factors in the regression models in text. Especially, age and possibly gender would affect the main outcomes substantially, so the results in Tables 2&3 might consider including these controlled demographic factors.

**Author’s response**: We thank the reviewer for this comment. Although social desirability may occur in surveys, it was not of concern in this study as the fieldworkers were not acquaintances to the participants.

Although the age range of the participants is between nine and eighteen, the questions were simple and easy to understand by the children. Our pilot study showed that the working children had a higher level of maturity than expected for their age considering the responsibilities they assume as breadwinners in support of their families.

We added the following sentence in the limitations:

In addition, the study did not use age-specific measures to capture the indicators in the study population. Despite their age range (>8-18 years), the pilot study did not reveal problems in the children’s understanding of the questions. The working children in this marginalized community have taken responsibilities at an early age, and tend to be more mature for their age.

Both age and gender were controlled for in the regression models, we added this in the manuscript. It is also included in a footnote underneath tables 2 and 3.

**Comment 5**: P.20: The results in the second paragraph is something age-sensitive and yet authors haven't explained this factor at all and treated age as if something neutral and insignificant. Another main flaw of the study is too narrowly defined and assessed "family social capital", which is defined too individualistically and not accurately reflecting family-level social capital.
Limitations can be more expanded with some of the above-mentioned comments. Implications can also include more specific suggestions for policy and practice on the ground.

**Author’s response**: We thank the reviewer for this comment. Our study population included children between 8 and 18 years. Although our analysis did not stratify by age group. Our observations and the pilot testing among the various age groups (>8 to 18 years) showed that the children had a good comprehension and clear perception of their connectedness with their social environment and their surroundings. This special group of working children were more mature than their age. They are working children with family responsibilities; this common burden of supporting their family has made them behave and think older than their age.

We added the following sentence in the limitations:

In addition, the study did not use age-specific measures to capture the indicators in the study population. Despite their age range (>8-18 years), the pilot study did not reveal problems in the children’s understanding of the questions. The working children in this marginalized community have taken responsibilities at an early age, and tend to be more mature for their age.

Regarding the definition of family social capital, we would like to point out that we have used the definition of Coleman because it is specific to children since our study population consisted of children. This definition was mentioned

Family social capital is identified by Coleman [24] as consisting of the relations between children and their parents or other family members, and as dependent on the quality of their relations to children [29].

Based on the reviewer’s comment we have added this clarification to the Section Questionnaire and measures section, subsection Social capital measures:

In addition, we have included in our analysis another aspect of social capital relevant to children, which is family social capital that includes the quality of relations between children and their parents [29].

We have added the following suggestions for policy and practice in the Conclusions section:

Multidimensional interventions that consider social capital dimensions ~~are needed~~ might contribute to improving the health of Syrian refugee children in informal tented settlements. Such interventions could include setting up peer-to-peer support programs that promote emotional wellbeing among child refugees. Other initiatives include the creation of spaces where children can play and socialize, enhancing social connectedness in their community. In addition, organizing social gatherings that bring together Syrian refugees and members of the host communities can promote more interaction, cooperation, and solidarity for the refugee children and their families.

**REVIEWER 3 COMMENTS**

Abstract

**Comment 1:** Please include age of the children.

**Author’s response**: The age of the children interviewed in this study is mentioned in the Methods and Findings section of the Abstract in the following sentence:

Children (>8 to 18 years) gave direct testimony on their living and social environment in face-to-face interviews.

Methods

**Comment 2:** Two references to the study authors 'recently published work' (page 6)are the same/listed twice in the reference list (reference 4 & reference 52).

**Author’s response**: We thank the reviewer for drawing our attention to this repetition. We have omitted the duplicate reference (number 52) and made the necessary edits to the in-text citations and the reference list.

**Comment 3:** What information is contained in the Interagency Mapping Platform as the database used for sampling?

**Author’s response**: We have added details on the Interagency Mapping Platform database, which was used for sampling, in the Methods section as follows:

The IAMP is a database used for the coordination of humanitarian activities. It contains information such as the number of Syrian refugee ITSs, their location, and the number of tents and individuals within each settlement.

**Comment 4:** Can one assume that you interviewed all children that met the eligibility criteria in the household (with consent)? Reporting the number of children per household would be helpful to provide some context for the analysis where you have adjusted for effect of clustering at household level.

**Author’s response**: All working children between 8 and 18 years in the surveyed households were interviewed. Oral informed consent was obtained from the female homemaker in the household and assent was obtained from the interviewed children.

We have added the average number of working children per household in Table 1 and in the first paragraph of the Results section:

On average, households housed 6.7 members including 2.8 working children.

**Comment 5:** What languages were the interviews conducted in? Was information collected on the literacy levels of the children? Completed schooling appropriate to their age? Please detail all socio-demographic data collected.

**Author’s response**: The interviews were conducted in colloquial Arabic. We have added this information to the Methods section:

The interviews were conducted in colloquial Arabic and took place at the participants’ households with the working children.

We have included information on the children’s school attendance and whether they attended classes appropriate to their age or were lagging behind in Table 1 and in the Results section, paragraph 1:

About 83% of the working children were not going to school. Out of those who were attending school (around 17%), only 16% were in grades appropriate to their age; while 84% were lagging behind in their education. This lag in age to school grade may have been the consequence of the children’s engagement in work activities.

**Table 1. Sociodemographic characteristics of the working children (>8-≤18 years) in 1,902 Syrian refugee households living in informal tented settlements, Bekaa, Lebanon, 2017 (N=4,090).**

| Characteristics | N | % |
| --- | --- | --- |
|  |  |  |
| ~~Age, years (Mean, SD)~~ | ~~13.2~~ | ~~2.7~~ |
|  |  |  |
| Age, years |  |  |
| 8-10 | 663 | 16.2 |
| 11-12 | 819 | 20 |
| 13-16 | 1860 | 45.5 |
| 17-18 | 748 | 18.3 |
|  |  |  |
| Gender |  |  |
| Male | 2107 | 51.5 |
| Female | 1983 | 48.5 |
|  |  |  |
| Field of work^a^ |  |  |
| Agriculture | 3098 | 75.8 |
| Waste picking | 173 | 4.2 |
| Construction | 147 | 3.6 |
| Car wash | 100 | 2.4 |
| Street services | 98 | 2.4 |
| Factory employees | 87 | 2.1 |
| Mechanics | 78 | 1.9 |
| Other^b^ | 382 | 9.3 |
|  |  |  |
| Going to school |  |  |
| Yes | 702 | 17.2 |
| No | 3388 | 82.8 |
|  |  |  |
| Child over age for grade^c,d^ |  |  |
| Yes | 589 | 83.9 |
| No | 113 | 16.1 |
|  |  |  |
|  | **Mean** | **SD^e^** |
|  |  |  |
| Number of children/household | 2.8 | 1.5 |
|  |  |  |
| Hours worked daily ~~(Mean, SD)~~ | 6.7 | 3 |
|  |  |  |
| Income/month, USD^f^ ~~(Mean, SD)~~ | 72.04 | 59.97 |

^a^ Total greater than 100% as more than one option is possible.

^b^ Includes handcrafts, packaging at markets, sewing, housekeeping, porting and other occupations.

^c^ Out of 702 children who go to school.

^d^ Based on age/school grade distribution in Lebanon: UNHCR & GoL. (2019). Education Sector. In: Lebanon Crisis Response Plan 2017-2020. Retrieved from:  <https://www.un.org.lb/library/assets/Education-015415.pdf>

^e^ Standard Deviation.

^f^ United States Dollar.

**Comment 6:** Were fieldworkers recruited on the basis of their experience of working/research with young people and/or their language skills?

**Author’s response**: The fieldworkers were recruited in association with a Lebanese NGO that has a history of working with Syrian refugees in different areas in Lebanon, including the Bekaa. In addition, this NGO has worked on addressing issues related to child health and protection. All the fieldworkers were native Arabic speakers. We have added this information to the Methods section as follows:

Fieldworkers were recruited through a Lebanese NGO that works with Syrian refugees in Lebanon, including those in the Bekaa, and whose activities include addressing issues related to child health and protection. All fieldworkers were native Arabic speakers. They attended a seven-day training workshop prior to beginning the fieldwork. The interviews were conducted in colloquial Arabic and took place at the participants’ households with the working children.

**Comment 7:** The detailed list of questionnaire items and measures in the text could be complemented by including the entire interview schedule/questionnaire in an appendix.

**Author’s response**: We thank the reviewer for this comment. We have included the requested information in Supplementary file 3, Questions and Answer Choices.

**Comment 8:** Was any piloting of the interview questions undertaken?

**Author’s response**: We thank the reviewer for this question. The interview questions were piloted in an area that was not selected in the study sample. We have added information on the pilot study in the Methods section, paragraph 2:

A pilot study was carried out in an ITS that was not selected in the final study sample. This allowed for testing the questionnaire and introducing adjustments to adapt the questions to the study population.

Results

**Comment 9:** Table 1: age, hours worked daily and income (mean, standard deviation) require different column headings than n,%

**Author’s response**: We thank the reviewer for this comment. We have edited Table 1 and added different column headings for the variables for which we reported Mean and SD:

**Table 1. Sociodemographic characteristics of the working children (>8-≤18 years) in 1,902 Syrian refugee households living in informal tented settlements, Bekaa, Lebanon, 2017 (N=4,090).**

| Characteristics | N | % |
| --- | --- | --- |
|  |  |  |
| ~~Age, years (Mean, SD)~~ | ~~13.2~~ | ~~2.7~~ |
|  |  |  |
| Age, years |  |  |
| 8-10 | 663 | 16.2 |
| 11-12 | 819 | 20 |
| 13-16 | 1860 | 45.5 |
| 17-18 | 748 | 18.3 |
|  |  |  |
| Gender |  |  |
| Male | 2107 | 51.5 |
| Female | 1983 | 48.5 |
|  |  |  |
| Field of work^a^ |  |  |
| Agriculture | 3098 | 75.8 |
| Waste picking | 173 | 4.2 |
| Construction | 147 | 3.6 |
| Car wash | 100 | 2.4 |
| Street services | 98 | 2.4 |
| Factory employees | 87 | 2.1 |
| Mechanics | 78 | 1.9 |
| Other^b^ | 382 | 9.3 |
|  |  |  |
| Going to school |  |  |
| Yes | 702 | 17.2 |
| No | 3388 | 82.8 |
|  |  |  |
| Child over age for grade^c,d^ |  |  |
| Yes | 589 | 83.9 |
| No | 113 | 16.1 |
|  |  |  |
|  | **Mean** | **SD^e^** |
|  |  |  |
| Number of children/household | 2.8 | 1.5 |
|  |  |  |
| Hours worked daily ~~(Mean, SD)~~ | 6.7 | 3 |
|  |  |  |
| Income/month, USD^f^ ~~(Mean, SD)~~ | 72.04 | 59.97 |

^a^ Total greater than 100% as more than one option is possible.

^b^ Includes handcrafts, packaging at markets, sewing, housekeeping, porting and other occupations.

^c^ Out of 702 children who go to school.

^d^ Based on age/school grade distribution in Lebanon: UNHCR & GoL. (2019). Education Sector. In: Lebanon Crisis Response Plan 2017-2020. Retrieved from:  <https://www.un.org.lb/library/assets/Education-015415.pdf>

^e^ Standard Deviation.

^f^ United States Dollar.

**Comment 10:** This reviewer's preference is to see the unadjusted Odds Ratios presented; these could be included in tables as supplementary files.

**Author’s response**: We thank the reviewer for this comment. We have created tables with unadjusted Odds Ratios and included them as supplementary files (S4 Unadjusted Odds Ratios) as requested by the reviewer.

**Comment 11:** It is best to avoid repeating the AdjOR and CI values in the text when these are detailed in the tables.

**Author’s response**: We thank the reviewer for this comment. We have abbreviated the Adjusted Odds Ratio term to AOR to be concise and included this information in the text for accuracy.

**Comment 12:** Typo at the bottom of Table 3 (page 19) with the number '40'?

**Author’s response**: the number 40 that shows at the bottom of table 3 is a line number since the word document has Line Numbers.

Discussion

**Comment 13:** Typo page 20, line 49 'spending' instead of 'sending'

**Author’s response**: We thank the reviewer for pointing out this typo. We have corrected it:

Although higher income may translate to greater access to health-supporting resources and improving health outcomes, it also implies that children are spending more time at work and hence less time with friends, which can lead to greater loneliness and less life satisfaction [57].

**Comment 14:** Reference is made on page 22 to the challenge in the analysis in terms of homogeneity of households in terms of housing problems and poverty conditions. Some details earlier about household composition, household income, housing and food insecurity would be useful in placing this limitation into context.

**Author’s response**: We thank the reviewer for this comment. We have added information on the household composition, household income, housing and food insecurity in the Results section, paragraph 1:

On average, households housed 6.7 members including 2.8 working children. The monthly household income per capita was 50.7 USD and the majority of households (74.3%) were severely food insecure [51].

**Comment 15:** The authors have an opportunity here to articulate what the potential multidimensional interventions could be. What role do (or can) existing social, humanitarian, health service play in innovative approaches to strengthening the social capital and cohesion of working children in these and other settlements? Did the authors hear from the participating children as to their perspectives about what strategies would help them?

**Author’s response**: We thank the reviewer for this comment. We have added the following recommendations on possible interventions that might strengthen the social capital and social cohesion of the working children living in these settlements:

Multidimensional interventions that consider social capital dimensions ~~are needed~~ might contribute to improving the health of Syrian refugee children in informal tented settlements. Such interventions could include setting up peer-to-peer support programs that promote emotional wellbeing among child refugees. Other initiatives include the creation of spaces where children can play and socialize, enhancing social connectedness in the community. In addition, organizing social gatherings that bring together Syrian refugees and members of the host communities can promote more interaction, cooperation, and solidarity for the refugee children and their families.

We did not ask the children on their perspectives about what strategies could help them.
