# Supplementary material for: Social capital, social cohesion, and health of Syrian refugee working children living in informal tented settlements in Lebanon: A cross-sectional study
Source: PLoS Med. 2020 Sep 2;17(9):e1003283. doi: 10.1371/journal.pmed.1003283 (PMC7467280; doi:10.1371/journal.pmed.1003283)
Supplement: Attachment — Submitted filename: Draft7-Response to Reviewers-SocialCapital-18July20.docx [file pmed.1003283.s008.docx]

**REQUESTS FROM THE EDITORS**

**Comment 1:** Please do include your response regarding the questionnaire validity as a limitation of the study, in the Discussion section and in the limitations of the Abstract.

**Author’s response**: Thank you. We have added the following to the Discussion section and in the limitations of the Abstract:

In the Abstract:

The main limitations of this study were its cross-sectional design as well as other design issues (using self-reported health measures, using a questionnaire that was not subject to a validation study, and giving equal weighting to all the components of the health and emotional wellbeing indicators).

In the Discussion:

Further, the study had design limitations that may have affected the findings. Specifically, the survey questionnaire was not subject to a validation study. However, the questionnaire items have been commonly used in the social capital and health literature, and the pilot study showed that the children understood the questions and responded clearly. In addition, self-reports of health may be unreliable given the study population’s limited access to adequate health care services that identify health problems in children.

**Comment 2:** Please do incorporate your response to the reviewer regarding the study's novelty into the discussion.

**Author’s response**: We thank the editor for this comment. We have edited the Discussion section as follows:

The social capital elements in this study were associated with refugee working children’s health. We also highlight the ~~critical~~ potential role of social cohesion in establishing and maintaining social connectedness that is related to positive health. The findings ~~It~~ also highlight~~s~~ the relationship ~~importance~~ of social capital ~~in creating a ripple effect of promoting~~ with belonging and social inclusion. Although the associations between social capital and health have been well discussed in the literature, there is a dearth of research on the interplay between these factors among Syrian refugees in humanitarian settings. This paper focused on the interconnectedness between social capital dimensions and the health of Syrian refugee children. These relationships have not been well addressed among Syrian refugee children facing precarious living and working conditions, which is a main contribution of this study.

**Comment 3:** Please do address reviewer 3’s comments where appropriate in the Discussion section.

**Author’s response**: We have addressed all comments and revised the Discussion section accordingly.

**Comment 4:** Data availability statement: Please revise your statement and include data access and contact information. You have noted in your response that “For enquiries about the project and data, please contact Ms. H. Mansour at the Faculty of Health Sciences, American University of Beirut. Email: hm102@aub.edu.lb.” However, please note that the contact point for granting data access cannot be one of the study’s authors.

You also note that “All relevant data are within the manuscript and its Supporting Information files.” PLOS defines the “minimal data set” to consist of the data set used to reach the conclusions drawn in the manuscript with related metadata and methods, and any additional data required to replicate the reported study findings in their entirety. Authors do not need to submit their entire data set, or the raw data collected during an investigation, but please submit the values behind the means, standard deviations and other measures reported.

**Author’s response**: We have modified the statement “All relevant data are within the manuscript and its Supporting Information files” and included the statement on Data Availability as below:

“Due to ethical issues surrounding this highly vulnerable study population living in a sensitive context (attitudes, hostility sometimes from surrounding communities, children and perhaps parents working with a non-documented status), exposing their data is a potential risk to their safety and wellbeing. Consequently, the data from the study cannot be made public. The data underlying the results are available, upon request, from the Department of Environmental Health at the Faculty of Health Sciences, American University of Beirut. For enquiries about the project and data, please contact [envd@aub.edu.lb](mailto:envd@aub.edu.lb)”.

Please not that the email provided in the statement above is an institutional email and is not for one of the authors.

**Comment 5:** Prospective analysis plan: Did your study have a prospective protocol or analysis plan? Please state this (either way) early in the Methods section.

a) If a prospective analysis plan (from your funding proposal, IRB or other ethics committee submission, study protocol, or other planning document written before analyzing the data) was used in designing the study, please include the relevant prospectively written document with your revised manuscript as a Supporting Information file to be published alongside your study, and cite it in the Methods section. A legend for this file should be included at the end of your manuscript.

b) If no such document exists, please make sure that the Methods section transparently describes when analyses were planned, and when/why any data-driven changes to analyses took place.

c) In either case, changes in the analysis-- including those made in response to peer review comments-- should be identified as such in the Methods section of the paper, with rationale.

**Author’s response**: We have a prospective analysis plan as part of the concept note that was written before analyzing the data and used in designing the study. The concept note has been added to the revised manuscript as a Supporting Information file and cited in the Methods section as indicated below. The assigned numbers of the other Supporting Information files and the legend has been modified accordingly.

The study is based on a detailed protocol that was used in designing the study (see S1 Text. Study Protocol). The detailed study methodology is also reported in recently published work [4, 6, 50, 51].

**Comment 6:** Abstract: Methods and Findings: Please change “(>8 to 18 years)” to “(8 to 18 years)” for the ages of the children.

**Author’s response**: We have changed the phrase about the ages of children in the Methods and Findings section in the Abstract as follows:

Children (8-18 years) gave direct testimony on their living and social environment in face-to-face interviews.

**Comment 7:** Abstract: Methods and Findings: For the associations between family social capital and social support and reporting more satisfaction with life, please report exact p values rather than p<0.04.

**Author’s response**: As per the editor’s request, we have added in the Methods and Findings section in the Abstract the exact p-value (p=0.04) for the associations between family social capital and social support and reporting more satisfaction with life as follows:

… more satisfied with life [(AOR: 1.3, CI: 1.01-1.54, p=0.04), (AOR: 1.2, CI: 1.01-1.4, p=0.04)

**Comment 8:** Author summary: Why was this study done?: Please revise to reduce the number of bullet points, as follows:

--The war in Syria has resulted in a large displacement of the population to neighboring

Lebanon, where many vulnerable displaced families are living in informal tented settlements with no access to means of livelihood and resources and child labor among these displaced families is high.

--This study aimed to investigate the associations between social capital and cohesion and

the health and emotional well-being of Syrian refugee children.

**Author’s response**: Thank you. We have updated this section of the Author summary as suggested by the editor. The section now reads as follows:

Why was this study done?

- The war in Syria has resulted in a large displacement of population to neighboring Lebanon, where many vulnerable displaced families are living in informal tented settlements ~~and have~~ with no access to means of livelihood and resources, and child labor among these displaced families is high.
- This study aimed to investigate the associations between social capital and cohesion and the health and emotional wellbeing of Syrian refugee children.
- ~~Results from this study help inform policies and strategies that improve the wellbeing of these child refugees.~~

**Comment 9:** Author summary: What did the researchers do and find?: Please revise to reduce the number of bullet points, as follows:

--We conducted a cross-sectional study of 1,902 households of Syrian displaced families

living in informal tented settlements in a rural area in Lebanon, and interviewed 4,090 working Syrian refugee children aged between 8 and 18 years.

--Around 11% of the working children rated their health as poor, 16% reported having a

physical health problem, and 13% were engaged in risky health behaviors.

--Lower social cohesion was significantly associated with reporting poor health, and a lower level of social support was significantly associated with engaging in risky health behaviors.

--Higher levels of neighborhood attachment, family social capital, and social support were significantly associated with greater optimism and life satisfaction, and higher levels of neighborhood attachment and social cohesion were significantly associated with feeling less lonely.

**Author’s response**: Thank you. We have updated this section of the Author summary as suggested by the editor as follows:

What did the researchers do and find?

- We conducted a cross-sectional study of 1,902 households of Syrian displaced families living in informal tented settlements in a rural area in Lebanon, and ~~We~~ interviewed 4,090 working Syrian refugee children aged between 8 and 18 years.
- Around 11% of the working children rated their health as poor, 16% reported having a physical health problem, and 13% were engaged in risky health behaviors.
- Lower social cohesion was significantly associated with reporting poor health, and a lower level of social support was significantly associated with engaging in risky health behaviors.
- Higher levels of neighborhood attachment, family social capital, and social support were significantly associated with greater optimism and life satisfaction, and higher levels of neighborhood attachment and social cohesion were significantly associated with feeling less lonely.

**Comment 10:** Introduction: Please remove section sub-headers from the Introduction.

**Author’s response**: We have removed all section sub-headers from the Introduction.

**Comment 11:** Introduction: bottom of page 5: Instead of bullet points, please summarize the definition of social capital in the most relevant way for your study (in paragraph form).

**Author’s response**: Thank you. We have revised the definitions and changed into paragraph form as follows:

~~Across these conceptual disputes, however, three foundational definitions of social capital are commonly acknowledged:~~ Social capital The main aspects of social capital that are highlighted in the literature include how it is produced through interaction ~~and it~~ resulting in material and symbolic profits for members of a group [23], ~~Social capital is embedded~~ its integration in everyday interactions between people in the form of obligations, expectations, information channels, and norms that encourage some practices and sanction others [24], and how it ~~Social capital refers to~~“facilitates coordination for mutual benefit” through “features of social organization, such as networks, norms, and trust~~, that facilitate coordination and cooperation for mutual benefit~~” [25].

**Comment 12:** Methods: First paragraph: Please remove the strike-through text for citation 52.

**Author’s response**: Thank you. We have removed the strike-through text for citation 52.

**Comment 13:** Methods: Please specify the nature of informed consent, and can you please clarify if the “female homemaker” was the children’s parent or guardian (or other relationship).

**Author’s response**: We have clarified this as follows:

We obtained oral informed consent from the female homemaker, who was often the children’s parent, and assent from the working children. If the female homemaker was not available, we interviewed an adult household member.

**Comment 14:** Methods: top of page 10: Please clarify what is meant by SC15, and if an abbreviation please spell it out in the text somewhere “‘Do you have a close friend in the neighborhood?” (SC15)”

**Author’s response**: We have removed this typo from page 10 as follows:

The question used for this indicator in the study is ‘Do you have a close friend in the neighborhood?” ~~(SC15)~~;

**Comment 15:** Methods: Statistical analysis: Please mention the factors adjusted for in your analyses.

**Author’s response**: The following information is presented in the Statistical analysis section of the Methods:

The relationships between the outcome variables and exposures were assessed using logistic regression models adjusted for district of residence, gender, and age of the household member, and adjusting for the effect of clustering at the household level.

**Comment 16:** Results: first paragraph, page 12: The following sentence seems speculative, and might be more appropriate in the discussion, unless you are presenting supporting data in the paper: “This lag in age to school grade may have been the consequence of the children’s engagement in work activities.”

**Author’s response**: We agree with this suggestion and have removed this sentence from the Results section as follows:

About 83% of the working children were not going to school. Out of those who were attending school (around 17%), only 16% were in grades appropriate to their age; while 84% were lagging behind in their education. ~~This lag in age to school grade may have been the consequence of the children’s engagement in work activities.~~

**Comment 17:** Results page 14: Socioeconomic characteristics: for the relationships between income and health and loneliness measures, please also include the unadjusted OR, or note the table where they are presented.

**Author’s response**: The unadjusted odds ratios are included as footnotes for Tables 2 and 3 as follows:

^b^ Adjusted Odds Ratio – please see supplementary file (S5 Unadjusted Odds Ratios) for corresponding table with unadjusted odds ratios).

**Comment 18:** Results: page 18: In the section on Optimism and Life Satisfaction, please report the exact p value for the relationship between social relations and satisfaction with life, rather than “p<0.04”.

**Author’s response**: We have added the exact p-value (p=0.04) for the relationship between social relations and satisfaction with life:

… being more satisfied with life [(AOR: 1.3, CI: 1.01-1.54, p=0.04)

**Comment 19:** Discussion: first paragraph: Please replace the word “strongly” with “significantly” if that is your intended meaning. Otherwise, please delete the word “strongly” as it is a subjective term.

**Author’s response**: We have replaced the word “strongly” with “significantly” in paragraph 1 of the Discussion. The sentence now reads as follows:

Social capital and social cohesion were ~~strongly~~ significantly associated with the health measures examined in this study.

**Comment 20:** Discussion: third paragraph: Please revise the first sentence of this paragraph to: “In this study, we found that a higher level of social cohesion is associated with better health for refugee working children” or similar.

**Author’s response**: We have replaced the sentence in paragraph 3 of the Discussion with the sentence suggested by the editor as follows:

~~Our findings showed that a higher level of social cohesion was associated with better health for refugee working children.~~ In this study, we found that a higher level of social cohesion is associated with better health for refugee working children.

**Comment 21:** Discussion: Middle of page 24: Please revise the following sentences to: “Indeed, a lower level of neighborhood attachment was associated with engaging in risky behaviors and loneliness, while being highly attached to their neighborhood showed associations with optimism and life satisfaction.” or similar, to clarify.

**Author’s response**: Thank you for this suggestion. We have edited these sentences in the Discussion as suggested by the editor.

Indeed, a lower level of neighborhood attachment was associated with engaging in risky behaviors and loneliness, while being highly attached to their neighborhood showed ~~strong~~ associations with optimism and life satisfaction.

**Comment 22:** Discussion: Please remove the sub-headings “Limitations” and “Implications”

**Author’s response**: We have removed both sub-headings from the Discussion.

**Comment 23:** Discussion: As mentioned by the reviewer, on page 25 please clarify the sentence “The working children in this marginalized community have taken responsibilities at an early age, and tend to be more mature for their age.” indicating what is meant by “more mature” and any supporting references.

**Author’s response**: We have revised this sentence as follows:

The working children in this marginalized community have taken responsibilities at an early age~~, and tend to be more mature for their age~~ and have had a wider exposure to the issues addressed in this study than what can be expected of children in their age.

**Comment 24:** Discussion: Please revise the following sentences in the Implications paragraph to avoid implying causality. We suggest: “We also highlight the potential role of social cohesion in establishing and maintaining social connectedness that is related to positive health. The findings also highlight the relationship of social capital with belonging and social inclusion.”

**Author’s response**: Thank you for this comment. We have modified this sentence based the editor’s suggestion as follows:

We also highlight the ~~critical~~ potential role of social cohesion in establishing and maintaining social connectedness that is related to positive health. The findings ~~It~~ also highlight the relationship ~~importance~~ of social capital ~~in creating a ripple effect of promoting~~ with belonging and social inclusion.

**Comment 25:** Discussion: Conclusions: Please revise the following sentence in the Conclusion paragraph: “In spite of the poor living and working conditions that Syrian refugee children encounter, those who enjoy a closely-knit network of family and friends reported better health than those who did not.”

**Author’s response**: We have revised this sentence as follows:

In spite of the poor living and working conditions that Syrian refugee children encounter, ~~those who enjoy~~ having a closely-knit network of family and friends ~~have experienced~~ was associated with better health. ~~than those who did not~~

**Comment 26:** Discussion: Conclusions: Please keep the conclusions focused on the main points of the study’s findings. The following sentences seem more appropriate for the paragraph discussing implications and future directions: “Multidimensional interventions that consider social capital dimensions might contribute to improving the health of Syrian refugee children in informal tented settlements. Such interventions could include setting up peer-to-peer support programs that promote emotional wellbeing among child refugees. Other initiatives include the creation of spaces where children can play and socialize, enhancing social connectedness in the community. In addition, organizing social gatherings that bring together Syrian refugees and members of the host communities can promote more interaction, cooperation, and solidarity for the refugee children and their families.”

**Author’s response**: Thank you for this comment. We agree and have moved this sentence to the paragraph discussing implications and future directions.

**Comment 27:** Table 1: Please remove the strikethrough text.

**Author’s response**: Thank you. We removed the strikethrough text in Table 1.

**Comment 28:** Figure 1: Is this figure taken directly from Carpiano, 2007?- if so we may not be able to include it due to copyright issues.

**Author’s response**: Thank you for alerting us to this copyright issue. Figure 1 has been removed from the manuscript as requested by the editor.

**Comment 29:** S1 Figure (map): Please confirm that you are able to freely share this image under CC BY 4.0 (In particular, given the text from <https://eur03.safelinks.protection.outlook.com/?url=https%3A%2F%2Fscholarworks.aub.edu.lb%2Fhandle%2F10938%2F21507&amp;data=02%7C01%7Crima.habib%40aub.edu.lb%7Ccaa908363e89426df7b808d81ed705ee%7Cc7ba5b1a41b643e9a1206ff654ada137%7C1%7C0%7C637293256149010046&amp;sdata=xks%2B9y28V2KfFaw%2BoGNvnApFCmcfU2RXTxNUSM1NY9w%3D&amp;reserved=0:> “Copyright Statement
All digitized texts and images in the AUB Libraries collections are for the personal, not-for-profit use of students, scholars, and the public. Any such use must name The American University of Beirut Libraries as the original source for the material. All texts and images are subject to copyright laws and, except where noted otherwise, are the property of the University Libraries. Commercial use, print or electronic re-publication of text or images (including reposting on the web an integral text or image) is strictly prohibited without prior written permission from the University Libraries. Reproduction Service Fee information is available on our website.”)

**Author’s response**: We received the below comment from the production office, indicating that the image does not breach copyright. We have cited the Journal of Public Health as the source:

1. Habib RR, Ziadee M, Abi Younes E, El Asmar K, Jawad M. The association between living conditions and health among Syrian refugee children in informal tented settlements in Lebanon. Journal of Public Health. 2019.

**Comment 30:** S4 file (supporting information Table 2 and Table 3): Please include descriptive legends for both tables, and please define abbreviations for USD, CI, and OR within the legend.

**Author’s response**: Thank you. We have added legends for both tables, and defined the abbreviations for USD, CI, and OR within legends as follows:

^a^ Model clustered at household level with unadjusted odds ratios.

^b^ Unadjusted Odds Ratio.

^c^ Confidence Interval.

^d^ United States Dollar.

**Comment 31:** STROBE checklist: Thank you for including the checklist. There are two question marks that need to be replaced with location information for reporting participant numbers and unadjusted results, under “Participants” and “Main Results”.

**Author’s response**: We thank the reviewer for drawing our attention to this. We have replaced the question marks with location information for reporting participant numbers and unadjusted results as follows:

Results, paragraph 1

S1 Table and S2 Table for the Unadjusted Odds Ratios

**REVIEWER 1 COMMENTS**

**Comment 1**: Thanks authors for their effort to improve the manuscript. I am mostly satisfied with the response and the revision. However, the response to my question on the validity of the questionnaire (comment 1) should be included in the limitation in the discussion of the paper. Also, the response to the novelty of the study (comment 3) should be included and highlighted in the discussion. This is because both questions are very important and of interesting to all the readers.

**Author’s response**: We thank the reviewer for this comment. As indicated earlier, we have edited the Discussion in accordance with reviewer’s comments as follows:

For comment 1 on validity of the questionnaire:

Further, the study had design limitations that may have affected the findings. Specifically, the survey questionnaire was not subject to a validation study. However, the questionnaire items have been commonly used in the social capital and health literature and the pilot study showed that the children understood the questions and responded clearly. In addition, self-reports of health may be unreliable given the study population’s limited access to adequate health care services that identify health problems in children.

For comment 3 on novelty of the study:

The social capital elements in this study were associated with refugee working children’s health. We also highlight the ~~critical~~ potential role of social cohesion in establishing and maintaining social connectedness that is related to positive health. The findings ~~It~~ also highlight the relationship ~~importance~~ of social capital ~~in creating a ripple effect of promoting~~ with belonging and social inclusion. Although the associations between social capital and health have been well discussed in the literature, there is a dearth of research on the interplay between these factors among Syrian refugees in humanitarian settings. This paper focused on the interconnectedness between social capital dimensions and the health of Syrian refugee children. These relationships have not been well addressed among Syrian refugee children facing precarious living and working conditions, which is a main contribution of this study.

**REVIEWER 3 COMMENTS**

Thank you to the authors for their careful and thoughtful response to the reviewers' suggestions. I have two remaining comments:

**Comment 1:** In response the age range of the children and capacity of the younger children to respond to the questions, the authors note that the children are mature for their age. I would be somewhat cautious about making this statement as maturity can be considered as a multi-dimensional concept. It is striking the proportion of young working children including those as young as eight; and the level of minimal/no schooling. The finding in relation to schooling should be highlighted, and a comment made on how this may relate to social capital and social cohesion.

**Author’s response**: We agree with the reviewer that the concept of maturity is a complex one and should be applied carefully to this population of children. We have modified our statement in the limitations section as follows:

Despite their age range (8-18 years), the pilot study did not reveal problems in the children’s understanding of the questions. The working children in this marginalized community have taken responsibilities at an early age~~, and tend to be more mature for their age~~ and have had a wider exposure to the issues addressed in this study than what can be expected of children in their age.

Regarding the lack of schooling in young children and its relation to social capital and social cohesion, we have added the following statement to the discussion:

In addition, the fact that many of these working children do not have access to adequate schooling is likely to affect their current and future prospects. Education is an important component of social capital as it is a means to access information and resources and to create better future opportunities. Moreover, an educational environment, such as a school or a learning center, can provide the space for people (both children and parents) to meet, participate in shared activities, and create relationships that promote social cohesion in a community.

**Comment 2:** Thank you for the inclusion of the paragraph of how these findings could be used to inform innovation to build and support social capital in these communities. I did wonder if the authors could consider going beyond this by commenting on the need to focus on 'up-stream' factors to reduce the burden on children, enable engagement in schooling, and address the likely intergenerational impacts of poverty for these refugee children and their families.

**Author’s response**: Thank you, we have incorporated the following to reflect on upstream factors:

Considerations of social capital as a public policy tool to achieve social cohesion need to take into account the alternative conceptions of social capital rooted in an understanding of the refugees’ contexts, displacement and social interactions. In addition, several up-stream factors such as the role of neighborhoods and communities, role of institutions, and social and economic policies need to be considered to reduce the burden on children, enable engagement in schooling, and address the likely intergenerational impacts of poverty for the refugee children and their families.

**REQUESTS FROM THE PRODUCTION OFFICE**

**FIGURES and GRAPHICS:**

1. Please provide all main figures in .tif or .eps format. For more information about how to convert your figure files, please see our guidelines: <https://eur03.safelinks.protection.outlook.com/?url=http%3A%2F%2Fjournals.plos.org%2Fplosbiology%2Fs%2Ffigures&amp;data=02%7C01%7Crima.habib%40aub.edu.lb%7Cfd8384e9a73f414487a808d821bc5a80%7Cc7ba5b1a41b643e9a1206ff654ada137%7C1%7C0%7C637296440140195122&amp;sdata=2prs10J9GKARlujWk9zqltg%2BTUihCX8Mj7ZQ0GPzcv8%3D&amp;reserved=0>. Once you've converted your files to .tif or .eps, please also make sure that your figures meet our format requirements: <https://eur03.safelinks.protection.outlook.com/?url=http%3A%2F%2Fjournals.plos.org%2Fplosbiology%2Fs%2Ffigures%23loc-file-requirements&amp;data=02%7C01%7Crima.habib%40aub.edu.lb%7Cfd8384e9a73f414487a808d821bc5a80%7Cc7ba5b1a41b643e9a1206ff654ada137%7C1%7C0%7C637296440140195122&amp;sdata=QwRwn9fbYwfxa08zkDo%2Bi9Mo6pE07A7%2FcISUaUZ9%2F%2Fs%3D&amp;reserved=0> / <https://eur03.safelinks.protection.outlook.com/?url=https%3A%2F%2Fjournals.plos.org%2Fplosmedicine%2Fs%2Ffigures%23loc-how-to-submit-figures-and-captions&amp;data=02%7C01%7Crima.habib%40aub.edu.lb%7Cfd8384e9a73f414487a808d821bc5a80%7Cc7ba5b1a41b643e9a1206ff654ada137%7C1%7C0%7C637296440140195122&amp;sdata=8soBxEtc1F9Y73ktyDPbK7LXB1YPT8Y5ZaITxXFZ5Dc%3D&amp;reserved=0>

2. Please remove embedded figures from your manuscript file and ensure the most up to date versions of your figures are uploaded to our submission system as separate TIF or EPS files. Please do not remove the figure legends or captions from the manuscript.

3. Please ensure that all main figure files are clearly labeled in the File Inventory.

4. Please ensure that all main figure files are cited in ascending numerical order in the main text of the article.

**Author’s response**: Thank you, we have addressed all 4 comments on figures and graphics.

**METADATA:**

1. Please ensure your short title is correct in the submission form, including symbols, italicisation etc. Your short title will be typeset from the submission form directly. 

2. Please accurately complete the 'Competing Interests' section on our submission form, including any COIs declared by your co-authors. If you have no competing interests to declare, please add "The authors have declared that no competing interests exist". Otherwise please declare all competing interests beginning with the statement "I have read the journal's policy and the authors of this manuscript have the following competing interests:"

**Author’s response**: The 'Competing Interests' section will be accurately completed upon submission. The authors have declared that no competing interests exist.

3. In accordance with PLOS' data policy, please ensure that your Data Availability Statement in the submission form clearly identifies how readers can access your data. Data cannot be available on request, or only accessible by contacting one of the authors. Note that your Data Availability Statement will be typeset as it is written, so please ensure it is in complete sentences and appears as you would like it to in the published version.

For details on our data policy, please see <https://eur03.safelinks.protection.outlook.com/?url=http%3A%2F%2Fjournals.plos.org%2Fplosmedicine%2Fs%2Fdata-availability&amp;data=02%7C01%7Crima.habib%40aub.edu.lb%7Cfd8384e9a73f414487a808d821bc5a80%7Cc7ba5b1a41b643e9a1206ff654ada137%7C1%7C0%7C637296440140195122&amp;sdata=NKKcnBSmXXdEzUOYvrQwVEULvjuKdobMesm2%2BaMk0bs%3D&amp;reserved=0>

**Author’s response**: The Data Availability has been modified to the following:

“Due to ethical issues surrounding this highly vulnerable study population living in a sensitive context (attitudes, hostility sometimes from surrounding communities, children and perhaps parents working with a non-documented status), exposing their data is a potential risk to their safety and wellbeing. Consequently, the data from the study cannot be made public. The data underlying the results are available, upon request, from the Department of Environmental Health at the Faculty of Health Sciences, American University of Beirut. For enquiries about the project and data, please contact [envd@aub.edu.lb](mailto:envd@aub.edu.lb)”.

**ARTICLE FILE:**

1. Please indicate the corresponding author on the title page with an asterisk (*) and include the corresponding author's email address. Only an asterisk and the email address itself are required.

**Author’s response**: The corresponding author has an asterisk and the email address included in the title page.

2. Please ensure that your Ethics Statement is available in the Methods section of your manuscript in its entirety.
**Author’s response**: The complete ethics statement is available in the Methods section.

3. We have noted that you have the following present in your manuscript: List of keywords/ Financial Disclosure

We have removed these section from your manuscript file as this information is automatically pulled from the submission form. Please make sure that the information in the submission form is correct.
**Author’s response**: Thank you.

4. PLOS does not publish Appendices. Please incorporate them into the main manuscript, or upload each of your Appendices as a Supporting Information file in the form "S1 Text", “S2 Text” etc, and refer to it throughout the manuscript as such. For more information, please see <https://eur03.safelinks.protection.outlook.com/?url=http%3A%2F%2Fjournals.plos.org%2Fplosbiology%2Fs%2Fsupporting-information&amp;data=02%7C01%7Crima.habib%40aub.edu.lb%7Cfd8384e9a73f414487a808d821bc5a80%7Cc7ba5b1a41b643e9a1206ff654ada137%7C1%7C0%7C637296440140195122&amp;sdata=ezgxGTKYbDTb%2BQA9N2n0crPobkefzO8GnaT1Gek%2BMn4%3D&amp;reserved=0>. / <https://eur03.safelinks.protection.outlook.com/?url=https%3A%2F%2Fjournals.plos.org%2Fplosmedicine%2Fs%2Flatex&amp;data=02%7C01%7Crima.habib%40aub.edu.lb%7Cfd8384e9a73f414487a808d821bc5a80%7Cc7ba5b1a41b643e9a1206ff654ada137%7C1%7C0%7C637296440140195122&amp;sdata=mvHVQz%2FXt0MHzzybC2B06sZ2Y%2FCD7kY01dqw8wK4PQ8%3D&amp;reserved=0>

**Author’s response**: All appendices have been added as separate Supporting Information files.

**ACKNOWLEDGMENTS:**

1. Please remove the funding information from the Acknowledgments section of your manuscript file. The published version is pulled automatically from the “Financial Disclosure” field in our submission form and must not be duplicated. Please therefore take this opportunity to check that the information in this field is correct and completed in full.

**Author’s response**: We have removed funding information from the manuscript.

**REFERENCES:**

1. Please make sure all references are cited in ascending numerical order in the text.

**Author’s response**: All references have been checked and are now cited in ascending numerical order in the text.

**SUPPORTING INFORMATION:**

1. Thank you for providing the source for the map in S1 Fig. We are satisfied that the image does not breach copyright and we can publish it freely, can we please ask you to remove the S1-Map Caption from the file inventory and cite the Journal of Public Health as the source, as this is part of their requirement.
**Author’s response**: We have removed the Caption and cited the Journal of Public Health as the source in the file inventory.

Habib RR, Ziadee M, Abi Younes E, El Asmar K, Jawad M. The association between living conditions and health among Syrian refugee children in informal tented settlements in Lebanon. Journal of Public Health. 2019. doi: <https://doi.org/10.1093/pubmed/fdz108>

2. Please ensure your supporting information files are individually uploaded to the File Inventory as file type 'Supporting Information' and are not present in the manuscript file.

**Author’s response**: We have now upload the Supporting Information documents as separate files.

3. Please upload your Supporting Information items individually, rather than in a single file, as you refer to them individually in the text. Supporting Information files should be less than 10 MB each, and the individual figure files should be labelled clearly eg “S1 Fig”, "S1A Fig" etc.

**Author’s response**: We have now uploaded the Supporting Information individually.

4. "Please ensure that all Supporting Information files are clearly labeled in the file inventory. i.e. S1_Fig.tif, S1_Table.xlsx, S1_Text.doc, S1_Data.xlsx, S1_PRISMA_Checklist.docx, etc.

Each file type should be numbered starting at 1. Please also make sure that the file description matches the file name (e.g. S1_Fig.tif is listed as S1 Fig)."

**Author’s response**: We reviewed all labels to ensure clarity and consistency. We made sure that each file type is numbered starting at 1 and that the file description matches the file name according to PLOS Medicine guidelines, as provided in the example above: S1_Fig.tif is listed as S1 Fig.

5. Please ensure that each Supporting Information file has a legend at the end of the manuscript file, after the Reference list.

**Author’s response**: We have now included a legend for all Supporting Information files at the end of the manuscript.

6. Please note that your submission cannot contain any references to generic "supporting information" files and instead must refer to specific files using our naming convention (S1 Fig, S1 Table, S1 Text, S1 Data, etc.)

**Author’s response**: We refer to specific Supporting Information files in the manuscript using the Journal’s naming convention for example: S1 Fig.
